# Explainable hybrid deep learning framework with Grad-CAM for heartbeat-level arrhythmia classification

**DOI:** 10.3389/fphys.2026.1848316

**Published:** 2026-08-03

**Authors:** Sureshkumar Sundaramoorthy, Govardhan Karunanidhi

**Affiliations:** School of Electronics Engineering, Vellore Institute of Technology, Vellore, Tamilnadu, India

**Keywords:** 1D-CNN, arrhythmia classification, channel attention mechanism, deep learning, electrocardiogram, explainable AI, gated recurrent unit, Grad-CAM

## Abstract

Cardiac arrhythmia, a common sign of cardiovascular disease, is a leading cause of global mortality and morbidity. Timely and accurate detection of electrocardiogram (ECG) cardiac arrhythmia is essential for effective clinical intervention. Here, we present an explainable hybrid deep learning framework for automated ECG cardiac arrhythmia classification. The proposed model integrates one-dimensional convolutional neural network (1D-CNN) for local morphological features, a gated recurrent unit (GRU) to extract temporal dependencies in sequential ECG signals, and the channel attention technique to emphasize clinically important patterns. In addition, a gradient-weighted class activation mapping (Grad-CAM) module is integrated to enhance interpretability by highlighting complex portions of the ECG signals that influence model decision making. The proposed framework is evaluated on three benchmark datasets: Massachusetts Institute of Technology–Beth Israel Hospital (MIT-BIH) Arrhythmia, St. Petersburg INCART, and the MIT-BIH Supraventricular Arrhythmia Database (SVDB). The model achieves classification accuracy values of 99.69%, 99.73%, and 98.77% along with macro-F1 scores of 95.75%, 97.21%, and 94.58% and specificity values of 99.53%, 99.54%, and 98.63%, respectively. The experimental results demonstrated performance improvement of approximately 2%–5% over recent state-of-the-art techniques in terms of key performance metrics. These findings indicate that the proposed framework effectively integrates local morphological feature extraction with temporal modeling, providing a robust and scalable solution for ECG arrhythmia classification. Moreover, its computational efficiency and its use of single-lead ECG signals make it suitable for real-time deployment in wearable devices, remote monitoring systems, and resource-limited clinical settings.

## 1 Introduction

Cardiac arrhythmia is a prevalent manifestation of cardiovascular disease (CVD), characterized by abnormal heart rhythms such as atrial fibrillation, atrial flutter, ventricular fibrillation, bradycardia, and tachycardia, among other irregular heartbeat rhythms. As a significant contributor to global morbidity and mortality, CVD accounts for approximately 32% of annual deaths, corresponding to nearly 17.9 million fatalities annually. According to reports from the American Heart Association (AHA) and the Centers for Disease Control and Prevention (CDC), approximately one in three adults is affected by some cardiac disorder, emphasizing the urgent need for accurate diagnostic therapies (Di Cesare et al., 2024). Timely and accurate arrhythmia identification is critical to preventing life-threatening outcomes such as stroke, heart failure, and sudden cardiac arrest.

Among the various diagnostic modalities including echocardiography, cardiac catheterization, electrophysiology studies (EPS), and stress testing, electrocardiogram (ECG) is the most widely used diagnostic tool because of its non-invasiveness, affordability, and diagnostic reliability. However, manual ECG interpretation by clinicians is time-consuming and is subject to inter-observer variability (Bahrami and Fotouhi, 2025). Traditional machine learning (ML) algorithms such as decision tree (DT), support vector machine (SVM), random forest (RF), AdaBoost (adaptive boosting), and eXtreme gradient boosting (XGBoost), have been employed for ECG cardiac arrhythmia classification tasks (Sun et al., 2024). Although these techniques are effective in specific healthcare settings, they typically rely on handcrafted features and exhibit limited scalability, particularly on large-scale or imbalanced datasets. The advancement of deep learning (DL) has revolutionized ECG analysis by facilitating the end-to-end feature extraction directly from raw ECG signals (Anand et al., 2024). Convolutional neural networks (CNNs) show strong performance in extracting local morphological features from ECG signals (Shoughi et al., 2024), while recurrent neural networks (RNNs) comprising long short-term memory (LSTM), bidirectional LSTM (BiLSTM), and gated recurrent unit (GRU) are well suited for capturing temporal dependencies (Xiong et al., 2022). Pre-trained models, e.g., AlexNet and ResNet-50, are fine-tuned using optimized hyperparameters and achieve optimal classification results (Daydulo et al., 2023). A one-dimensional convolutional deep residual network utilizes the synthetic minority oversampling technique (SMOTE) to address the imbalanced class problem and effectively classifies cardiac arrhythmias, demonstrating superior performance on benchmark datasets such as Massachusetts Institute of Technology–Beth Israel Hospital (MIT-BIH) and INCART (Khan et al., 2023). In recent years, attention mechanisms integrated with CNN have further improved the classification accuracy and interpretation by allowing the models to focus on diagnostically pertinent portions of ECG data signals (Lamba et al., 2025). However, CNN models are intrinsically limited in their ability to capture long-range temporal features. A comparative study utilizing DL models, including one-dimensional CNN (1D-CNN), 2D-CNN, and CNN-LSTM, is suggested for automated cardiac arrhythmia detection using the MIT-BIH dataset, demonstrating enhanced performance over traditional DL techniques (Arora et al., 2023).

More recently, transformer-based models designed for natural language processing (NLP) have attained prominence in ECG signal analysis due to their capability to capture long-term dependencies and facilitate parallel computation. The hybrid DL networks CAT-Net (Islam et al., 2024) and ECG-TransCovNet, which integrate CNN with transformer to capture both local morphological features and temporal dependencies, achieved high accuracy and robust performance (Shah et al., 2024). However, these models often suffer from high computational complexity and are more suitable for large-scale datasets.

### 1.1 Related works

An accurate ECG arrhythmia classification is crucial in clinical practice as different cardiac arrhythmia types require distinct treatments. However, manual ECG analysis is challenging due to significant morphological variations. In recent years, DL approaches, particularly 1D-CNN and GRU, have shown strong performance in automated ECG arrhythmia classification using denoised ECG signals from benchmark databases such as MIT-BIH, INCART, and MIT-BIH Supraventricular Arrhythmia Database (SVDB). These models efficiently extract both temporal and morphological patterns, enabling a reliable and robust classification compared with conventional ML methods (Mahel et al., 2025). A hybrid residual network integrating standard and depthwise separable convolutions with residual connections was developed for ECG arrhythmia classification, achieving an accuracy of 99.09% on the MIT-BIH dataset while effectively handling class imbalance using SMOTE (Qi et al., 2024). A hybrid recurrent CNN optimized using grey wolf optimization (GWO) was developed for ECG arrhythmia classification, achieving an accuracy of 98%, outperforming conventional ML methods such as SVM, RF, and *k*-nearest neighbor (KNN) (Singh and Mahapatra, 2024). A stacked multi-model DL framework was introduced to minimize the training complexity and manual feature engineering, achieving 98.37% sensitivity, 99.59% specificity, 98.41% precision, and 99.35% accuracy (Irfan et al., 2022). Attention-enhanced CNN-LSTM models have achieved classification accuracy values exceeding 98% on multiclass arrhythmia detection tasks (Issa et al., 2025). Peng et al. (2024) proposed a dual-attention CNN-LSTM model, which had 88.76% accuracy but limited minority class performance. Yang et al. (2023) introduced a lightweight heart rate variability (HRV)-based model predicting sudden cardiac death (SCD), which had 91.17% accuracy. Both highlight attention and feature fusion for improved ECG diagnostics. Moreover, data imbalance poses significant challenges to model generalization and clinical reliability. Despite significant advancements in DL for cardiac arrhythmia detection, the development of a reliable and universally robust classification framework remains a critical challenge. Existing approaches often face challenges related to variability in ECG signal quality, model complexity, and generalization across diverse datasets. Furthermore, concerns regarding interpretability, robustness, and clinical validation continue to hinder the practical adoption of these models in real-world healthcare settings (Jaya Prakash et al., 2025).

**Figure 1:**
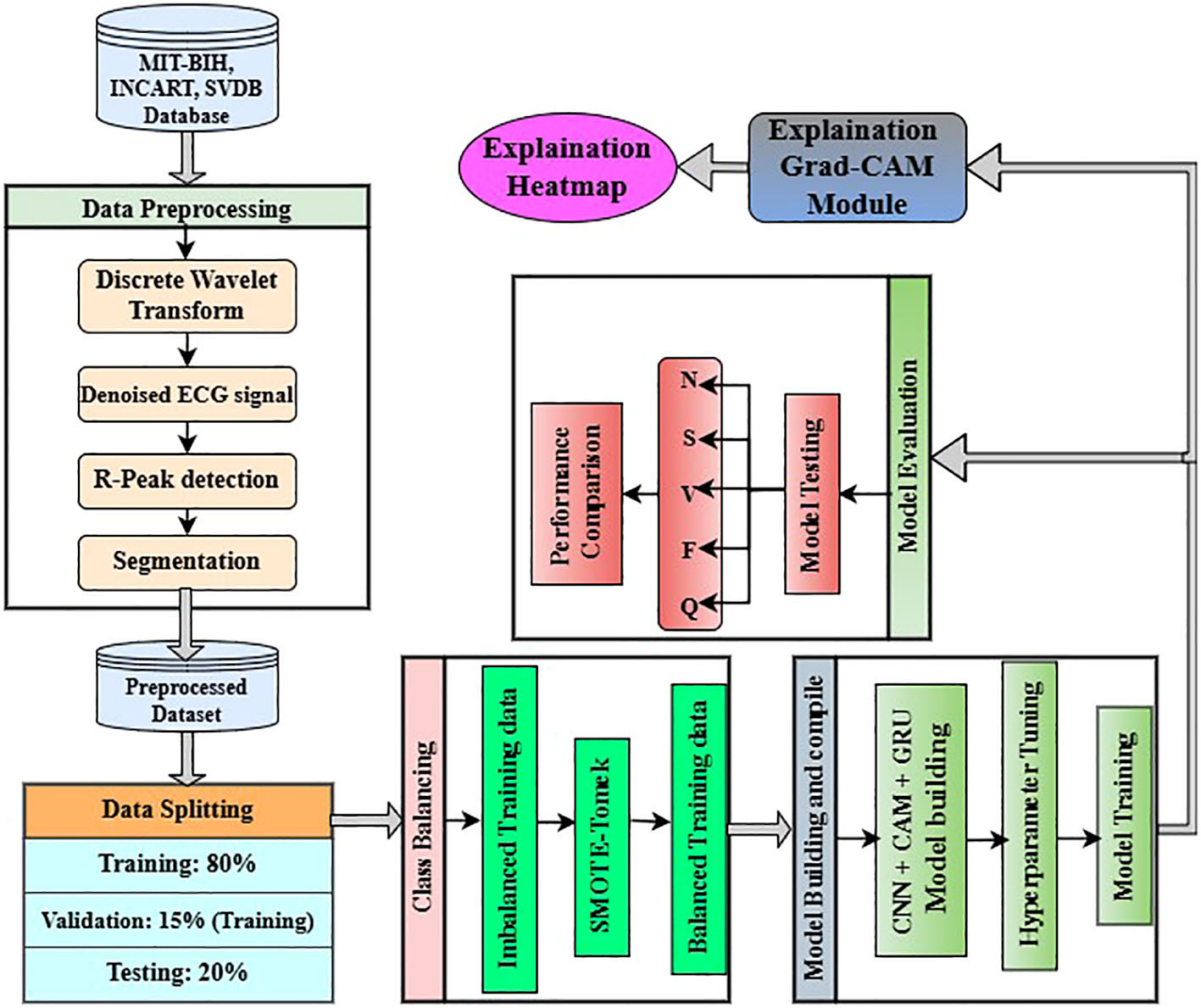
Outline of the proposed architecture framework.

To address these limitations, this work developed an explainable attention-based hybrid framework for multiclass ECG arrhythmia classification. The proposed framework comprises a CNN that extracts the ECG data signal local features, channel attention (CA) that increases training effectiveness by capturing the most informative temporal ECG segments, and a GRU that accurately captures temporal patterns and resolves problems such as vanishing and exploding gradients during lengthy sequence training. The proposed model was evaluated on three popular databases: the MIT-BIH Arrhythmia database, the INCART database, and the MIT-BIH SVDB. The study explores various DL architectures, including CNNs and RNNs, to establish a robust and efficient classification framework. The overall system architecture of the single-lead ECG cardiac arrhythmia detection model is shown in **Figure 1**. The main contributions of this work are summarized as follows:

1. An explainable attention-based CNN-GRU hybrid DL framework integrated with gradient-weighted class activation mapping (Grad-CAM) is developed for accurate multiclass ECG arrhythmia classification.
2. An effective ECG preprocessing pipeline is employed to remove baseline drift, powerline interference, myoelectric artifacts, and high-frequency artifacts along with amplitude normalization to enhance signal quality.
3. A segmentation strategy is employed to extract heartbeat segments with a fixed window size of 300 samples, focusing on informative regions of the ECG signal.
4. The SMOTE–Tomek technique, combining SMOTE and Tomek links, is used to improve minority class prediction by generating synthetic samples and removing overlapping instances, thereby refining class boundaries and enhancing the overall class balance in the proposed model.
5. Extensive validation is conducted through comparative analysis with multiple baseline CNN model variants and cross-dataset validation on the INCART and SVDB datasets, demonstrating improved robustness and generalization capability.

The explainable attention-based CNN-GRU hybrid DL framework and its hyperparameters were systematically optimized to conduct a thorough empirical evaluation. This optimization produced a highly efficient model, achieving accuracy values of 99.69%, 99.73%, and 98.77% on MIT-BIH, INCART, and SVDB, respectively. The remainder of this work is organized as follows. The dataset details, preprocessing pipelines, class balancing, proposed network design, and explainability module are illustrated *Section 2*. The evaluation metrics, classification performance, model hyperparameter tuning, computation complexity analysis, ablation studies, and comparison with other techniques are discussed in *Section 3*. Finally, *Section 5* summarizes our findings in the conclusion section.

## 2 Methodological framework

A description of the datasets employed, the data preprocessing strategies, and the class balancing approaches are outlined in this section. Subsequently, the proposed framework is presented, comprising three primary elements: CNN, CA, and GRU. The specifics of the Python implementation are also outlined in this section.

### 2.1 Dataset

The Association for the Advancement of Medical Instrumentation (AAMI) standard ECG databases such as MIT-BIH, St. Petersburg INCART 12-lead Arrhythmia, and the MIT-BIH SVDB were used in this work. Details about the datasets are presented in **Table 1**. **Table 2** provides the heartbeat counts of each ECG arrhythmia class.

**Table 1:** Details of the utilized datasets.

Content	INCART	MIT–BIH	MIT–BIH SVDB
No. of records	75	48	78
Instrument	Holter	Holter	Holter
Signals	12 leads (II lead signals used)	2 leads (ML-II and V1)	2 leads (ML-II and V1)
Time duration	30 min	30 min	30 min
Sampling frequency	257 Hz	360 Hz	128 Hz
Subjects	32 (15 women, 17 men)	47 (22 women, 25 men)	78 subjects
Age range	18–80 years	23–89 years	23–89 years
Heartbeat categories	11	15	11
Period	Not mentioned	1975–1979	1979
Laboratory	St. Petersburg Cardiological Laboratory	Beth Israel Hospital Laboratory	Beth Israel Hospital Laboratory

**Table 2:** Heartbeat counts of the Massachusetts Institute of Technology–Beth Israel Hospital (MIT-BIH) Supraventricular Arrhythmia Database (SVDB), INCART, and MIT-BIH datasets.

SN	Class	Heartbeat name	Heartbeat count		
			SVDB	INCART	MIT-BIH
1	Normal (N)	Normal (N) beat	161,985	150,087	74,920
2		Left bundle branch block (L) beat	–	1	8,063
3		Right bundle branch block (R)	1	3,164	7,244
4		Nodal (junctional) escape (j) beat	–	92	229
5		Atrial escape (e)	–	–	16
			-161,986	-153,344	-90,472
6	Supraventricular ectopic (S)	Atrial premature (A)	–	1,941	2,540
7		Aberrated atrial premature (a)	1	–	150
8		Non-conducted P-wave (n)	–	32	–
9		Nodal (junctional) premature (J)	9	–	83
10		Supraventricular premature (S)	12,175	16	2
			-12,185	-1,989	-2,775
11	Ventricular ectopic (V)	Premature ventricular contraction (V) Ventricular escape (E)	9,925	19,980	7,123
12			–	–	106
			-9,925	-19,980	-7,229
13	Fusion (F)	Fusion of normal and ventricular(F)	23	219	802
			-23	-219	-802
14	Unknown (Q)	Unclassifiable (Q)	79	6	33
15		Paced(/)	–	–	7,012
16		Fusion of paced and normal(f)	–	–	982
17		Signal quality change (|)	2,204	–	–
18		Noise/artifact (˜)	1,081	–	–
19		Rhythm change(+)	1	6	–
			-3,365	-12	-8,027
		Total heartbeats	-187,484	-175,544	-109,305

#### 2.1.1 MIT–Beth Israel Hospital Arrhythmia Database

There are 48 ECG records in the MIT–Beth Israel Hospital database (Moody and Mark, 2001), each recording with a time duration of 30 min. Between 1975 and 1979, MIT (Massachusetts Institute of Technology) and BIH (Beth Israel Hospital) collected these ECG recordings from 47 subjects. This database comprises 47 ECG signal recordings from patients of both genders (22 women and 25 men) aged between 23 and 89 years. Of the ECG recordings, 23 were randomly chosen from a total of 4,000 twenty-four-hour Holter ECG recordings collected from a mixed population of 40% outpatients and 60% inpatients at BIH, whereas 25 remaining samples were selected from the same set, but with clinically important arrhythmias. These ECG recordings were digitized at a sampling rate of 360 Hz, with an 11-bit resolution over a 10-mV amplitude range. Each record was independently annotated by two or more experienced cardiologists, and any discrepancies were resolved using reliable reference annotations. In total, the database comprises 109,305 annotated heartbeats. In our work, ML-II lead ECG signals were employed to build a single-lead ECG framework. This dataset comprises 23 unique heartbeat categories, and this research focused on a subset of 15 types, as presented in **Table 2**, which were grouped into five primary classes: normal (N), ventricular ectopic (V), supraventricular ectopic (S), fusion (F), and unknown (Q).

#### 2.1.2 St. Petersburg INCART 12-lead arrhythmia database

The St. Petersburg INCART 12-lead arrhythmia database contains 75 annotated ECG recordings from 32 Holter records, each ECG recording with time duration of 30 min. The ECG signals are acquired using 12 leads and sampled at a frequency of 257 Hz (Goldberger et al., 2000). The dataset includes recordings from 32 subjects (17 men and 15 women), aged between 18 and 80 years, primarily undergoing evaluation for coronary artery disease. The ECG recordings have a wide range of cardiac abnormalities, including supraventricular ectopy, atrial fibrillation, bundle branch block, and conduction disorders. This database comprises over 175,000 annotated heartbeats. For each record, detailed metadata such as patient demographics, clinical diagnoses, and ECG characteristics are provided. In this work, only the ML-II lead ECG data utilized from the 12-lead data were employed in our model. According to **Table 2**, the dataset comprises 11 annotated heartbeat categories, further grouped into five classes of arrhythmias, while fusion (F) beats and unknown (Q) beats are excluded, which only have 219 and 12 beats, respectively.

#### 2.1.3 MIT-BIH Supraventricular Arrhythmia database

The MIT-BIH SVDB (Moody and Mark, 2001) comprises 78 ECG recordings, each with a time duration of approximately 30 min, collected from patients exhibiting cardiac arrhythmias. The ECG signals are recorded using two leads (ML-II and V1) and sampled at a frequency of 128 Hz. The dataset primarily focuses on supraventricular ectopic activity and related arrhythmias, making it suitable for evaluating the generalization capability of classification models on atrial abnormalities. Each recording is annotated with beat-level labels. The annotated beats are categorized into five major classes (N, S, V, F, and Q), consistent with the AAMI standard classification, as presented in **Table 2**. The dataset comprises over 187,000 heartbeats with 11 annotated label categories, further grouped into five classes of arrhythmias, while fusion (F) beat is excluded, which only has 23 heartbeats. We begin by developing our model for four-class classification using the SVDB dataset, three-class arrhythmia classifications using INCART, and five-class classification using the MIT-BIH database and subsequently fine-tune it through class balancing, architectural modifications, hyperparameter tuning, and performance evaluation to assess its overall effectiveness and robustness.

### 2.2 Preprocessing data

**Figure 2:**
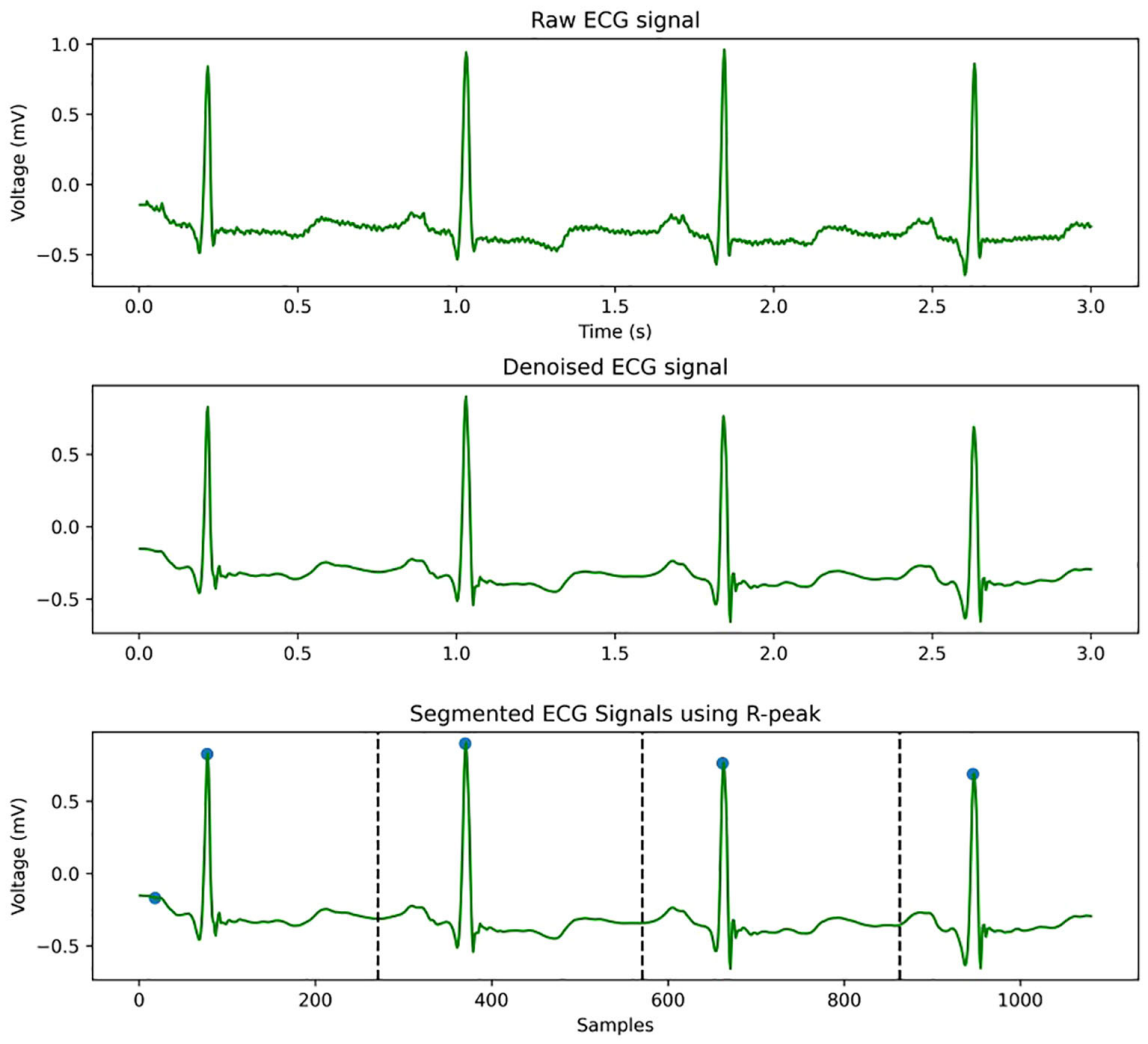
Visualization of raw, denoised, and R-peak annotated electrocardiogram (ECG) segments (record 100, ML-II lead, MIT-BIH dataset).

**Figure 3:**
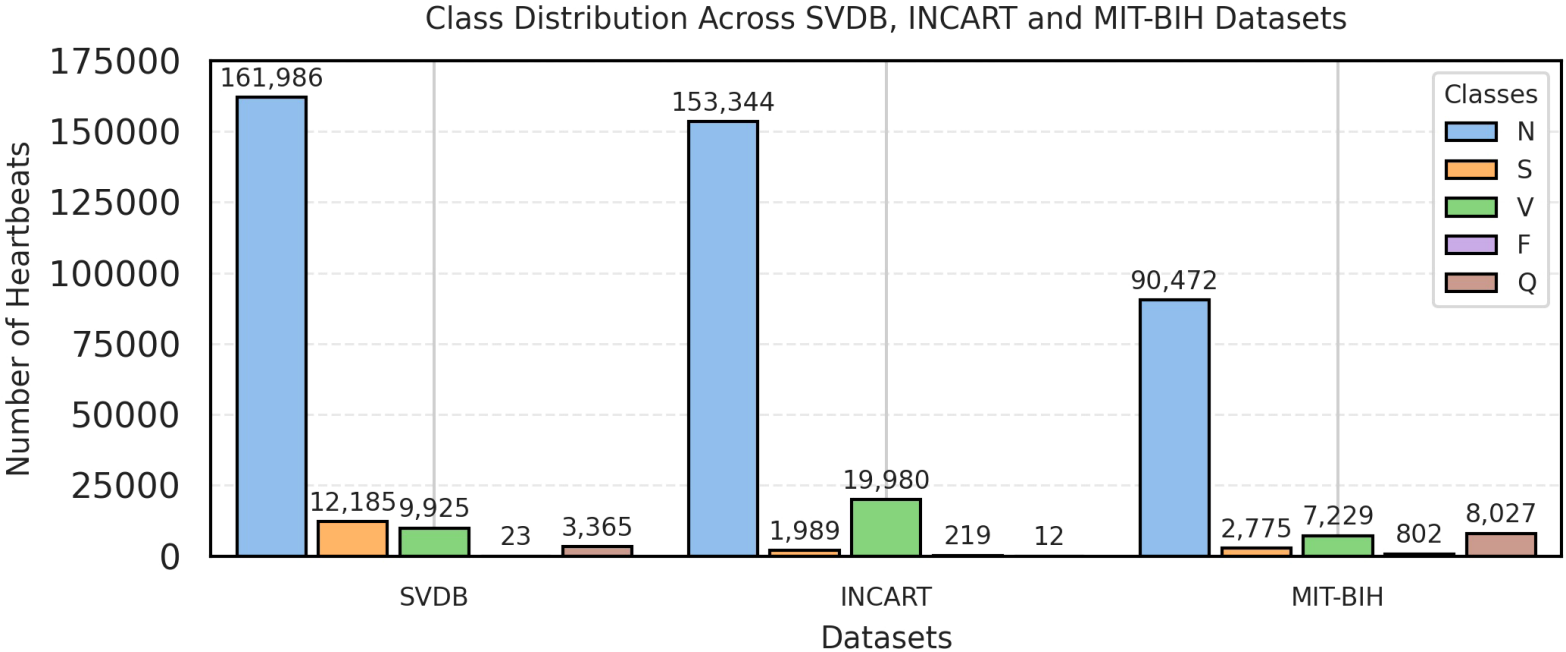
Class distribution across the Supraventricular Arrhythmia Database (SVDB), INCART, and MIT-BIH (Massachusetts Institute of Technology–Beth Israel Hospital) database.

The first step in preprocessing is to eliminate noise from the raw ECG signal. The second step is heartbeat segmentation from the denoised ECG signal. A number of noise sources, such as baseline drift, powerline interference, and myoelectric interference, corrupt the raw ECG signals. A popular technique for eliminating noise from the ECG signal is discrete wavelet transform (DWT). To remove noise from the ECG data, the wavelet denoising technique comprises three distinct processes: decomposition, coefficient processing, and wavelet reconstruction (Malleswari et al., 2024). The ECG data signal is initially decomposed into nine sublevels using the Daubechies (db5) wavelet, resulting in nine detail coefficients, from cD1 to cD9, and one approximate coefficient, cA9. The first two high-frequency detail coefficients, cD1 and cD2, which are related to the myoelectric artifact and powerline interferences, are completely suppressed. The mid-frequency coefficients from cD3 to cD6 comprise clinically important ECG components such as the P-wave, QRS complex, and ST-T segments. The lower-frequency coefficients (cD7–cD9) associated with baseline variations are processed using a soft-thresholding technique. The reconstructed ECG signal is obtained by applying the wavelet reconstruction technique to the denoised ECG signal using the processed approximate and detail coefficients. Once the signal is denoised, the ECG signal segmentation is then performed using the R-peak location. Artificial neural networks (ANNs), wavelet transforms, and the modified Pan-Tompkins algorithm are the techniques that have been outlined in the literature for identifying the R-peaks and segmenting the ECG signals (Tripathy and Pachori, 2024). The R-peak locations are already annotated in the INCART, MIT-BIH, and SVDB. Therefore, the fixed-window approach and the R-peak annotation locations were used to segment continuous ECG recordings into fixed-length windows, where each segment represents an individual heartbeat. A window of 300 samples was chosen, with 99 samples prior and 201 samples after the R-peak locations. This formulation enables heartbeat-level classification by emphasizing the local morphological characteristics of the ECG signals within each segmented heartbeat. Following this segmentation, each heartbeat segment is treated as an individual sample for model training and evaluation. The train–test split is therefore performed at the beat level, which is widely used in recent ECG arrhythmia classification studies focusing on fine-grained morphological feature extraction (Islam et al., 2024; Peimankar et al., 2024; Shah et al., 2024; Yu et al., 2025). To further evaluate the generalization capability and mitigate potential bias, cross-dataset validation was performed by training the model on the MIT-BIH dataset and tested on the benchmarked INCART and SVDB datasets. As these datasets comprise recordings from different subjects, this evaluation inherently ensures inter-patient separation and provides a more reliable assessment of model robustness. The visualization of the raw, denoised, and R-peak annotated ECG segments (record 100, ML-II lead, MIT-BIH dataset) are shown in **Figure 2**. The total heartbeats in each class distributed across the SVDB, INCART, and MIT-BIH datasets are shown in **Figure 3**.

### 2.3 Class data balancing

As shown in **Table 3**, the datasets employed in this work have an imbalanced sample distribution across the cardiac arrhythmia classes. As a result, the DL algorithms were biased toward the majority sample class and underperformed when predicting minority class data. For this reason, we opted to use the SMOTE–Tomek technique to balance the classes in the dataset. We chose this technique for its outstanding performance. Batista et al. (2004) developed the SMOTE–Tomek technique, which offers enhancements to the SMOTE algorithm. The difference between SMOTE and SMOTE–Tomek is that the SMOTE sampling technique generates new synthetic samples instead of oversampling with replacement. In this technique, initially, the difference in minority class sample *x_i_* and any one of its nearest neighbors ${\bar{x}}_{i}$ is calculated and then this value is multiplied by *δ*. This multiplied value is added to selected *x_i_* samples to generate a new sample, *x*_new_, as shown in Equation 1.

**Table 3:** Number of heartbeats used for training and testing in the SVDB, INCART, and MIT-BIH datasets.

Class	Heartbeats			Training (80%)			Testing (20%)		
	SVDB	INCART	MIT-BIH	SVDB	INCART	MIT-BIH	SVDB	INCART	MIT-BIH
N	161,986	153,344	90,472	129,599	122,686	72,328	32,387	30,658	18,144
S	12,185	1,989	2,775	9,727	1,611	2,241	2,458	378	534
V	9,925	19,980	7,229	7,942	15,948	5,807	1,983	4,032	1,422
F	23	219	802	18	180	639	5	39	163
Q	3,365	12	8,027	2,701	10	6,429	664	2	1,598
Total	187,484	175,544	109,305	149,987	140,435	87,444	37,497	35,109	21,861

SVDB, MIT-BIH Supraventricular Arrhythmia Database; MIT-BIH, Massachusetts Institute of Technology–Beth Israel Hospital; N, normal; S, supraventricular ectopic; V, ventricular ectopic; F, fusion; Q, unknown.

(1) $$x_{\text{new}}=x_{i}+({\bar{x}}_{i}-x_{i})\times \delta 1$$

where the *δ* value ranges from 0 to 1. The SMOTE–Tomek technique combines upsampling through SMOTE, with downsampling achieved by eliminating Tomek links. The Tomek links technique is used to detect nearest neighbor pairs with several classes within a dataset. A Tomek link is a set of neighbor pairs with minimal Euclidean distance (*x_i_,x_j_*), where *x_i_* refers to the minority class and *x_j_* to the majority class.

SMOTE–Tomek has two distinct benefits. Initially, Tomek link efficiently eliminates synthetic noise occurrences generated by SMOTE, ensuring that the dataset is more reliable and clean in order to train the model. Furthermore, it provides a more balanced dataset by sequentially resolving the challenges in upsampling and undersampling. This evenly distributed arrhythmia class distribution improves the classifier ability in order to generalize efficiently across each class.

**Table 3** summarizes the number of training and testing samples for each class across the INCART, MIT-BIH, and SVDB datasets, along with the corresponding class-balancing strategy. As the N class contains significantly more samples than the other classes, random undersampling (RUS) was initially applied prior to implementation of the SMOTE–Tomek technique. To avoid potential data leakage, class-balancing techniques were applied only to the training dataset after train–test splitting. This strategy prevents overlap between the training and testing samples and ensures an unbiased evaluation of the proposed model performance. Finally, balanced training datasets were generated, with each class containing 50,000 samples.

### 2.4 Architecture model

**Figure 4:**
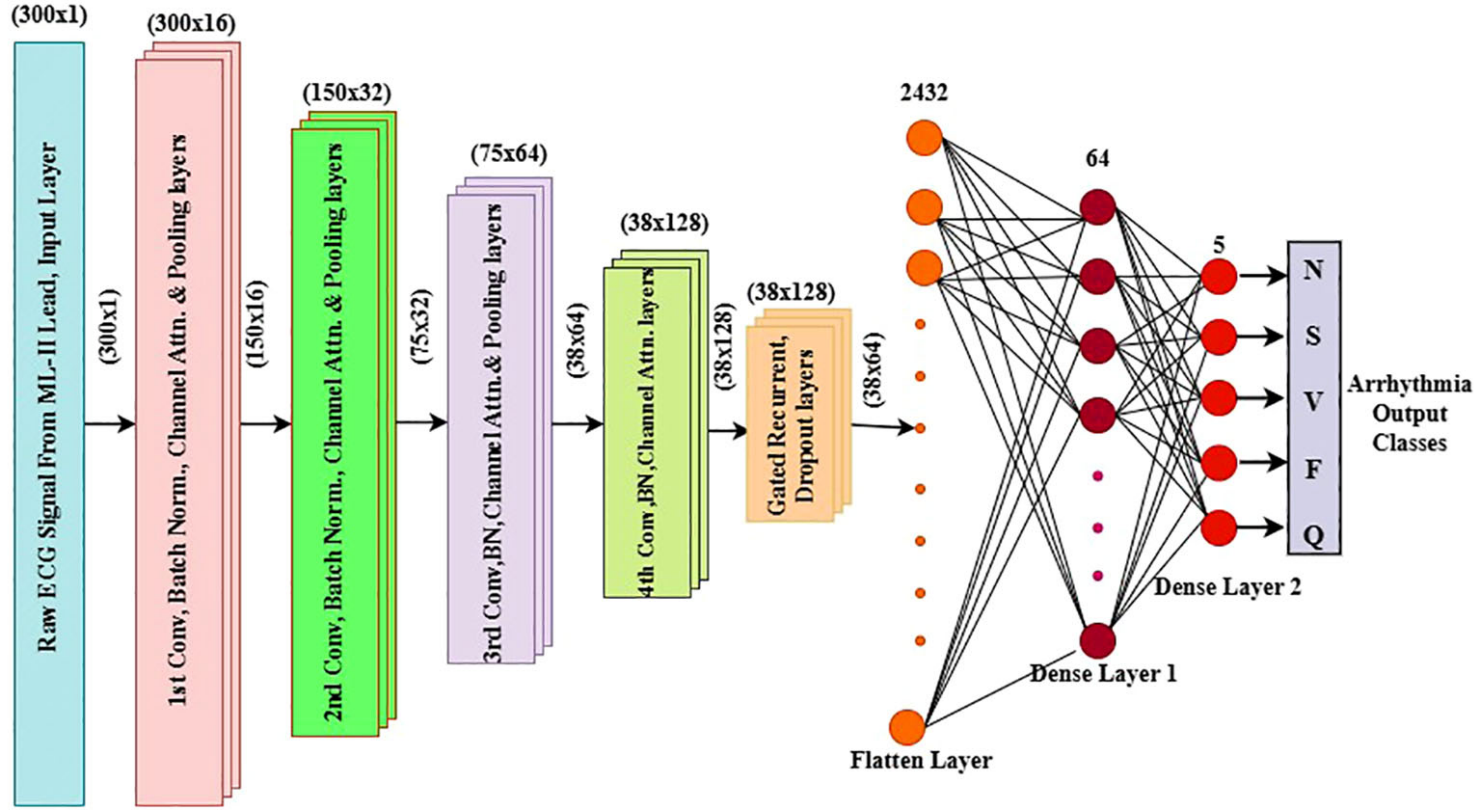
Proposed architecture for the classification of electrocardiogram (ECG) arrhythmias.

The proposed hybrid architecture comprises four key components: i) CNN, ii) CA, iii) GRU, and iv) an explainability (XAI) module, which is detailed in a separate subsection. The proposed model is implemented using the Keras application programming interface (API) with a TensorFlow backend. Each 1D convolutional layer employs the *same* padding with a stride of 1 and rectified linear unit (ReLU) activation, with convolutional blocks comprising 16, 32, 64, and 128 filters with corresponding kernel sizes of 21 × 1, 23 × 1, 25 × 1, and 27 × 1. Batch normalization is applied after each convolutional layer to stabilize training and accelerate convergence. The CA module is integrated after each convolutional block to enhance informative feature extraction. It utilizes both global average pooling and max pooling operations, followed by a shared multilayer perceptron (MLP) with a reduction ratio of *r* = 8 to effectively capture channel-wise dependencies. For temporal modeling, two GRU layers with 128 and 64 hidden units are employed. A dropout layer is used between the GRU layers to reduce overfitting and improve generalization. After feature extraction and temporal modeling, the resulting feature maps are flattened and passed through fully connected layers for multiclass arrhythmia classification using a softmax activation function. The proposed architecture network diagram is shown in **Figure 4**, and its summary is presented in **Table 4**.

**Table 4:** Proposed model summary.

Layer	No. of filters	Kernel size	Output dimension	Parameter
Conv1Dim	16	21 × 1	(None,300,16)	352
Batch Norm	–	–	(None,300,16)	64
Attn1Dim	16	–	(None,300,16)	0
Maxpool1Dim	–	3 × 1	(None,150,16)	0
Conv1Dim	32	23 × 1	(None,150,32)	11,808
Batch Norm	–	–	(None,150,32)	128
Attn1Dim	32	–	(None,150,32)	0
Maxpool1Dim	–	3 × 1	(None,75,32)	0
Conv1Dim	64	25 × 1	(None,75,64)	51,264
Batch Norm	–	–	(None,75,64)	256
Attn1Dim	64	–	(None,75,64)	0
Avgpool1Dim	–	3 × 1	(None,38,64)	0
Conv1Dim	128	27 × 1	(None,38,128)	221,312
Batch Norm	–	–	(None,38,128)	512
Attn1Dim	128	–	(None,38,128)	0
GRU	128	–	(None,38,128)	99,072
Dropout	–	–	(None,38,128)	0
GRU	64	–	(None,38,64)	37,248
Flatten	–	–	(None,2432)	0
Dense	–	–	(None,64)	155,712
Dropout	–	–	(None,64)	0
Dense	–	–	(None,5)	325
			Total	578,053

#### 2.4.1 CNN architecture

The effective performance of 1D-CNN in fields including medical imaging, computer vision, NLP, and autonomous vehicles has also translated into impressive outcomes in time-series data signal analysis such as ECG and electroencephalogram (EEG). 1D-CNN models have been employed to fix several difficult problems. Through convolution operations, local feature patterns within an ECG signal can be captured, which enables the generation of feature maps for subsequent layers (Mohebbanaaz et al., 2025). The proposed network described here has four CNN layers. The preprocessed ECG heartbeat segment of a fixed window length size of 300 × 1, extracted from the ML-II lead ECG recordings, is given as input to the 1D convolutional block for feature extraction. Raw ECG data are processed by the first 1D-CNN layer, and the feature map produced by the preceding layer is used as input by each subsequent layer. There are 16 filters of kernel size 21 × 1 with a single stride, and the same padding is employed in the first 1D-Conv (convolution) layer. Subsequently, the 1D max pooling layer has a size 3 × 1 and two strides are utilized. For the next three 1D-Conv layers, 32 filters of kernel size 23 × 1, 64 filters of kernel 25 × 1 size, and 128 filters of kernel size 27 × 1 are used, accordingly. Padding ‘same’ with one stride and the ReLU activation function are employed in all four 1D-Conv layers.

As the 1D-CNN goes deeper, both the counts of filters and the size of the kernel are progressively increased to allow the deeper layers to capture more complex features for precise prediction (Peng et al., 2024). To manage computing complexity, pooling layers are included in the first three 1D-Conv layers and the third 1D-Conv layer utilizing average pooling with 3 × 1 size, two strides, and ‘same’ padding. The average pooling is used after the third 1D-Conv layer instead of the max pooling layer because the deeper portion carries sensitive data.

The process for the four 1D-Conv layers can be illustrated as:

(2) $$x_{j}^{l}=\sigma (\sum_{i\in M_{j}}x_{i}^{l-1}\times \omega_{ij}+b_{j})$$

where $x_{j}^{l}$ denotes the output of the *j*-th neuron at layer l; $b_{j}$ denotes bias of the *j*-th neuron at layer l; $\omega_{ij}$ denotes the convolution kernel between the *i*-th and *j*-th neurons; σ indicates the activation function ‘ReLU’; and $M_{j}$ represents the convolution kernel effective range.

To keep the output mean close to zero and its standard deviation close to 1, a batch normalization (BN) layer is performed after each 1D-Conv layer. By accelerating fast convergence and stabilizing the training phase, this normalization minimizes the number of epochs needed to train deep networks efficiently. CNN has demonstrated superior ability in extracting local spatial patterns from ECG signals, making it suitable for arrhythmia classification tasks (Tripathy et al., 2019). The use of increasing kernel sizes and filters across deeper layers facilitates the learning of hierarchical features.

#### 2.4.2 Channel attention

**Figure 5:**
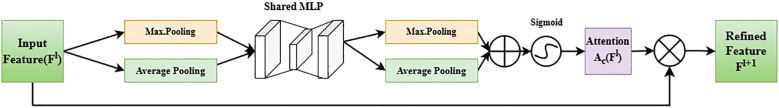
Channel attention mechanism for extracting the refined feature map.

In our hybrid model, CA is employed after every 1D-Conv layer. The integration of CA is aimed at increasing the training effectiveness by capturing the most informative temporal ECG segments. The proposed model used only single-lead data, generating a 2D feature map, and therefore only CA is used following the convolution block attention technique (Ezzat et al., 2024). The proposed CA schematic is presented in **Figure 5**. After every convolution, the 2D feature map is generated as $F\in {\mathbb{R}}^{C\times L}$, where L indicates the length and C denotes the number of channels. The 2D matrix feature map is initially transformed into the 1D matrix attention map, $A_{c}\in {\mathbb{R}}^{C\times 1}$, in the CA strategy. The attention-based refined $F^{l+1}$ feature map for the following layer is then generated by multiplying the 1D attention map $A_{c}$ with the $F^{l}$ feature map. By employing the element-wise matrix multiplication operator ⊗, the entire process is formalized in the *l*-th layer as $F^{l+1}=A_{c}(F^{l})⊗F^{l}$, where $F^{l}$ denotes the *l*-th layer feature map (Woo et al., 2018). CA is obtained in the spatial domain by aggregating the spatial data using maximum and average pooling. From this, two distinct spatial data presenters, $F_{c}^{\text{max}}$ and $F_{c}^{\text{avg}},\;$are produced. These presenters are given as input to the MLP with a single hidden layer. Thereafter, the feature vectors are generated using element-wise summation. Thus, attention mapping is generated in Equation 3.

(3) $$\begin{array}{c} A_{c}(F)=\sigma (\text{MLP}(\text{Maximum pool}(F))+\text{MLP}(\text{Average pool}(F)))\\ A_{c}=\sigma (W^{1}(W^{0}(F_{c}^{\text{max}}))+W^{1}(W^{0}(F_{c}^{\text{avg}}))) \end{array}$$

where $W^{0}\in {\mathbb{R}}^{\frac{C}{r}\times C}$, $W^{1}\in {\mathbb{R}}^{\frac{C}{r}\times C}$ are the weights of the MLP. In our model *r* = 8 is used to minimize variable overload. Therefore, the activation hidden size is ${\mathbb{R}}^{C\times 1\times 1}$. The output of the fourth CA layer is given to the GRU, which is discussed next. The 38 × 64 output of the GRU is then flattened into a vector size of 2,432. In the end, 64 dense-layer neurons evaluate this vector, and then a second dense layer has five neurons that utilize the activation function “softmax” to classify five distinct arrhythmia types.

#### 2.4.3 Gated recurrent unit

**Figure 6:**
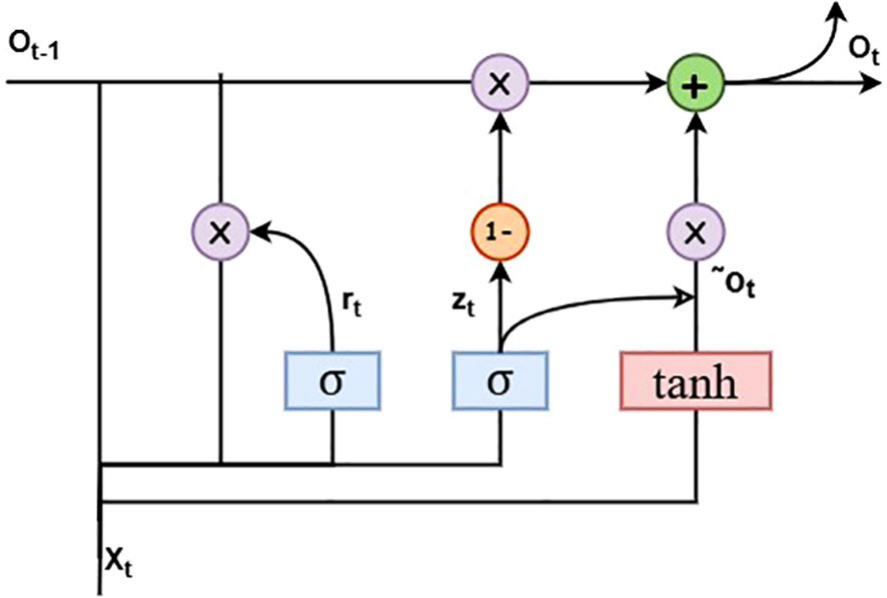
Gated recurrent unit architecture.

The 1D-CNN and CA layers that extracted the local spatial features are fed as the input to a GRU layer. 1D-CNN processes the input samples independently at each time step, as described by the CNN structure in Equation 2, mainly extracting spatial feature correlations. This method restricts their ability to fully portray the temporal characteristics of the time-series data (Peimankar et al., 2024). **Figure 6** shows the GRU architecture, which allows the output of each neuron at a specific time period to affect the output of the subsequent time periods. The sequential dependency of GRU enables accurately capturing temporal patterns and resolving problems such as vanishing and exploding gradients during the training of long sequences. The GRU employs two gating units, allowing it to determine the optimal combinations of new and previous information, as well as the extent of previous information retention required to calculate the new state, thus substantially reducing the computation during the time-consuming feature extraction process (Bai et al., 2024). The cell states and related outputs at each time step are outlined in Equations 4–7.

(4) $$r_{t}=\sigma (W_{xr}^{T}x_{t}+W_{or}^{T}o_{t-1}+b_{r}),$$

(5) $$z_{t}=\sigma (W_{xz}^{T}x_{t}+W_{oz}^{T}o_{t-1}+b_{z}),$$

(6) $${\tilde{o}}_{t}=\tan h\;(W_{x\tilde{o}}^{T}x_{t}+W_{o\tilde{o}}^{T}(r_{t}⊗o_{t-1})+b_{\tilde{o}}),$$

(7) $$o_{t}=z_{t}⊗o_{t-1}+(1-z_{t})⊗{\tilde{o}}_{t}.$$

where $W_{xz}$, $W_{xr}$, and $W_{x\tilde{o}}$ denote the weight matrices related to the current input $x_{t}$ for the update gate, reset gate, and candidate activation, respectively. Similarly, $W_{oz}$, $W_{or}$, and $W_{oeo}$ indicate the weight matrices associated with the hidden $o_{t-1}$ state from the prior time step. The vectors $b_{r}$, $b_{z}$, and $b_{\tilde{o}}$ are the respective bias terms.

### 2.5 Explainability (XAI) module

In DL-based medical applications, interpretability is essential to ensure that model predictions are reliable and clinically meaningful. This is particularly important in ECG signal analysis, where automated systems assist in diagnosing cardiac abnormalities. Despite achieving high performance, deep neural networks (DNNs) are often considered as black-box models, restricting their acceptance into clinical practice due to the lack of transparency in decision making. To address this limitation, the proposed framework integrates a Grad-CAM module to provide visual explanations of the model predictions. Grad-CAM is an efficient technique for the interpretation of DNNs by highlighting the regions of the signal that contributed most significantly to the classification decision (Selvaraju et al., 2020). In ECG signal analysis, this enables identification of the temporal segments that influence the detection of various cardiac arrhythmias.

The raw ECG signals from the MIT-BIH, INCART, and MIT-BIH SVDB datasets are preprocessed and segmented into fixed-length heartbeat windows of 300 samples (201 samples after and 99 samples prior) to each detected R-peak. This segmentation ensures temporal alignment of complex waveform components such as the P-wave, QRS complex, and T-wave. In addition, amplitude normalization is applied to minimize inter-patient variability and emphasize spatial–temporal features. The Grad-CAM generation process begins by extracting feature maps from the fourth and final 1D convolutional layer of the proposed model. Gradients of the target class score with respect to the feature maps are computed to identify the importance of each temporal segment. These gradients are globally averaged to produce weights, which are then combined with the feature maps to generate a coarse localization map. For a target class *c*, the Grad-CAM heatmap *M^c^* is calculated using Equation 8:

(8) $$M^{c}=\text{ReLU}(\sum_{k}\alpha_{k}^{c}A^{k})$$

where *A^k^* represents the *k*-th activation map of the final convolutional layer and $\alpha_{k}^{c}$ denotes the importance weight corresponding to class *c*. The importance weights are computed using global average pooling of gradients as shown in Equation 9:

(9) $$\alpha_{k}^{c}=\frac{1}{Z}\sum_{i}\sum_{j}\frac{\partial y^{c}}{\partial A_{ij}^{k}}$$

where *y^c^* denotes the target class score and *Z* represents the total number of elements in the feature map (Tenepalli and Navamani, 2025b). The resulting activation map is passed through a ReLU activation function and normalized to the range (0–1) for visualization. In this heatmap representation, higher values indicate regions that have a stronger influence on the model prediction.

The Grad-CAM heatmaps generated are overlaid on the related ECG signals to visually interpret the emphasis of the model. The results demonstrated that the model consistently attends to clinically relevant segment regions of the ECG signal. For example, high activation is observed around the QRS complex in cases of ventricular cardiac abnormalities, whereas the P-wave and T-wave regions are evident for other ECG arrhythmia types. This behavior aligns well with established clinical diagnostic criteria, where various waveform components indicate specific cardiac conditions.

By providing visual explanations, the Grad-CAM module improves the transparency and interpretability of the proposed hybrid architecture. It enables clinicians to verify whether the network is focusing on important physiological patterns, thus enhancing the trust in automated predictions. Furthermore, it aids in determining potential model limitations by revealing cases where attention may be directed toward less significant relevant regions. The integration of the Grad-CAM-based explainability module ensures that the proposed ECG classification architecture network not only achieves high predictive performance but also delivers interpretable and clinically consistent results. This significantly enhances its suitability in real-world clinical decision-making systems.

### 2.6 Experimental environment

The proposed model was executed in the Kaggle cloud-based environment with Python version 3.11. The ‘wfdb’ and ‘pywt’ (Waveform Database and PyWavelets) libraries were utilized for ECG data signal preprocessing and noise reduction. Model development and evaluation were carried out by utilizing TensorFlow and scikit-learn, with Keras functioning as the high-level API built on top of the TensorFlow engine. The experiments were conducted using Kaggle’s GPU runtime, which offered dual NVIDIA T4 GPUs, each with 16 GB of memory. The environment for computational purposes included an Intel Xeon processor, a 64-bit architecture, and CUDA-enabled acceleration, thus facilitating efficient training and evaluation of the DL models.

### 2.7 Training strategy and configuration

**Figure 7:**
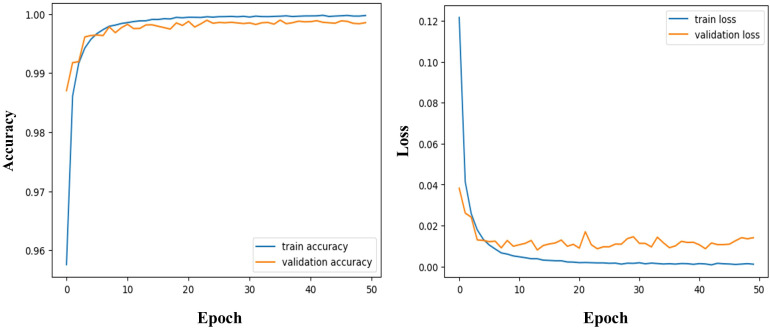
Accuracy and loss curves during training and validation of the proposed model using the Massachusetts Institute of Technology–Beth Israel Hospital (MIT-BIH) dataset.

**Figure 8:**
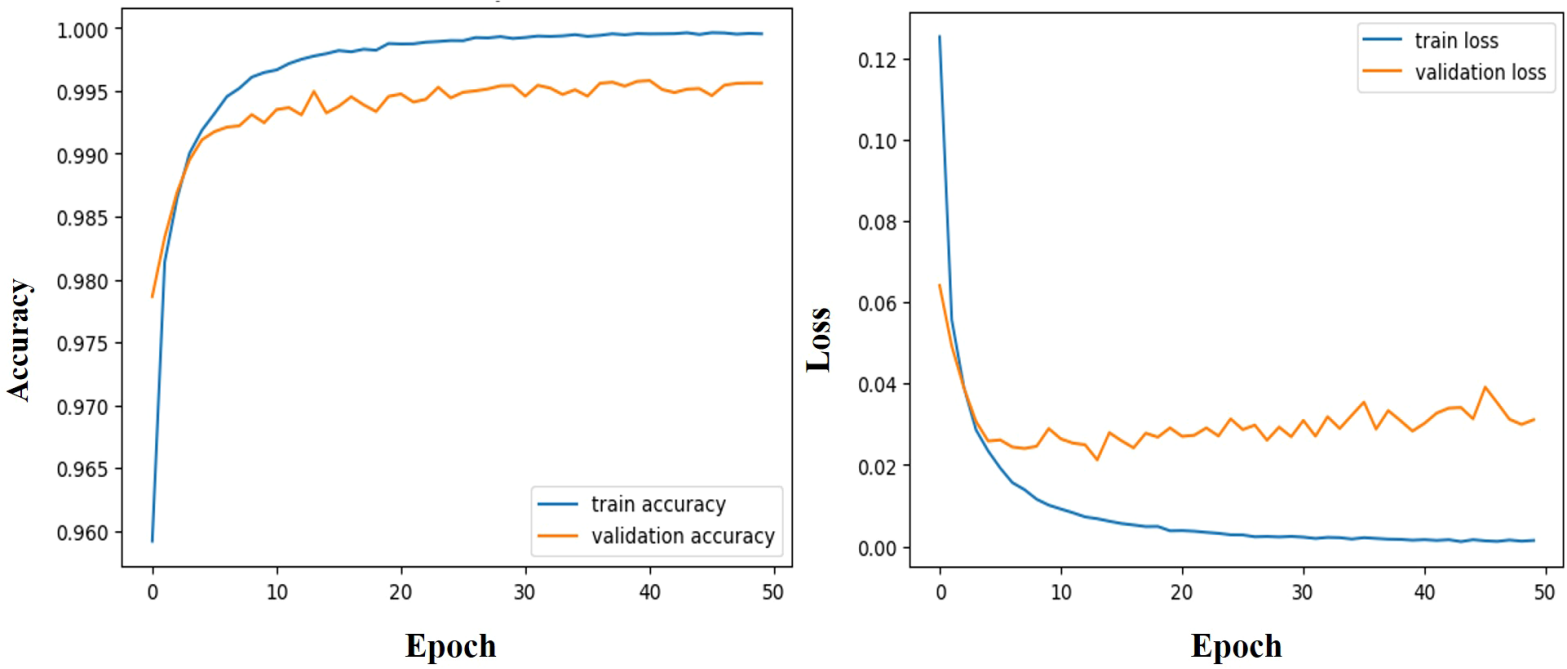
Accuracy and loss curves throughout training and validation of the proposed model using the MIT-BIH Supraventricular Arrhythmia Database (SVDB) dataset.

**Figure 9:**
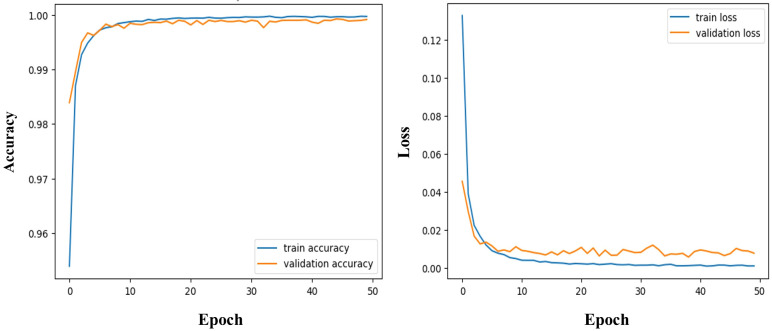
Accuracy and loss curves throughout training and validation of the proposed model using the INCART dataset.

The preprocessed ECG signal data were split into 80% for training and 20% for testing, with 15% of the training data used for validation. The model was trained over 50 epochs using mini-batch gradient descent with a batch size of 128. Optimization was performed using the ‘Adamax’ optimizer with default learning parameters. Model checkpointing was employed to preserve the optimal model weights based on validation accuracy, while TensorBoard was used to monitor the training progress. The accuracy and loss curves for training and validation across the MIT-BIH, SVDB, and INCART datasets are shown in **Figures 7**–**9**, respectively. The steady improvement in accuracy and the consistent reduction in loss indicate stable convergence and effective generalization. All experiments were conducted in a GPU-enabled Kaggle environment to ensure computational efficiency. These implementation settings ensure that the proposed framework can be reliably reproduced and evaluated in similar experimental conditions.

A progressive design strategy was employed during model development. The baseline CNN architecture was initially configured with four consecutive 1D-Conv layers comprising 16, 32, 64, and 128 filters. Subsequently, two GRU layers containing 64 and 128 units were added sequentially to enhance temporal feature extraction. The proposed hybrid model hyperparameter values are presented in **Table 5**.

**Table 5:** Proposed model hyperparameter values.

Hyperparameter	Value
Batch size	128
Epochs	50
CNN filter dimensions	16, 32, 64, 128
GRUs	64, 128
Dropout rate	0.02
Optimizer	Adamax
Validation split	15% (of training data)

## 3 Results and discussion

Several evaluation measures were used to evaluate and analyze the outcomes of the model. The evaluation metrics, classification performance, effects of changing the hyperparameters, computational complexity analysis, and proposed model validation are addressed below.

### 3.1 Evaluation metrics

The proposed hybrid model effectiveness was thoroughly evaluated with standard metrics comprising sensitivity, specificity, accuracy, and the F1 score. The overall correctness of the predictions is determined by accuracy, while sensitivity captured the model’s capability to identify true arrhythmic patterns, which is essential for medical diagnosis. The F1 score, as the harmonic mean of sensitivity and precision, facilitates a balanced view, which is particularly valuable in the presence of an imbalanced class. The area under the receiver operating characteristic curve (AUC-ROC) was employed to measure the model’s capability to distinguish different classes across several thresholds. These evaluation metrics were chosen to represent both the overall performance and the clinical importance of reducing diagnostic errors to ensure a thorough evaluation of the proposed hybrid model performance. Furthermore, the per-class and overall accuracy, sensitivity, specificity, precision, and macro-F1 scores were evaluated in accordance with Equations 10–15 as follows:

(10) $${\text{Sensitivity}}^{\text{PC}}={\text{Sensitivity}}^{\text{OA}}=\frac{\text{TP}}{\text{TP}+\text{FN}}$$

(11) $${\text{Specificity}}^{\text{PC}}={\text{Specificity}}^{\text{OA}}=\frac{\text{TN}}{\text{TN}+\text{FP}}$$

(12) $${\text{Precision}}^{\text{PC}}={\text{Precision}}^{\text{OA}}=\frac{\text{TP}}{\text{TP}+\text{FP}}$$

(13) $${\text{Accuracy}}^{\text{PC}}=\frac{\text{TP}+\text{TN}}{\text{TP}+\text{TN}+\text{FP}+\text{FN}}$$

(14) $${\text{Accuracy}}^{\text{OA}}=\frac{N_{\text{correct}}}{N}$$

(15) $$\text{Macro}-\text{F}1^{\text{OA}}=\frac{1}{C}\sum_{i=1}^{C}F1^{i,\text{PC}}$$

where true positive and negative and false positive and negative are denoted by TP, TN, FP, and FN, respectively (Yang et al., 2023). The overall outcome is denoted by OA, while the per-class performance is indicated by PC. The total number of samples and the correctly predicted samples are represented by *N* and *N*_correct_, respectively. The notation F1_OA_ signifies the overall F1 score, whereas the total classes is denoted by *C*. In addition, the F1 score for the *i*-th class is represented by F1*^i,^*^PC^, while *N_i_* indicates the total samples in the *i*-th class.

### 3.2 Classification performance

**Figure 10:**
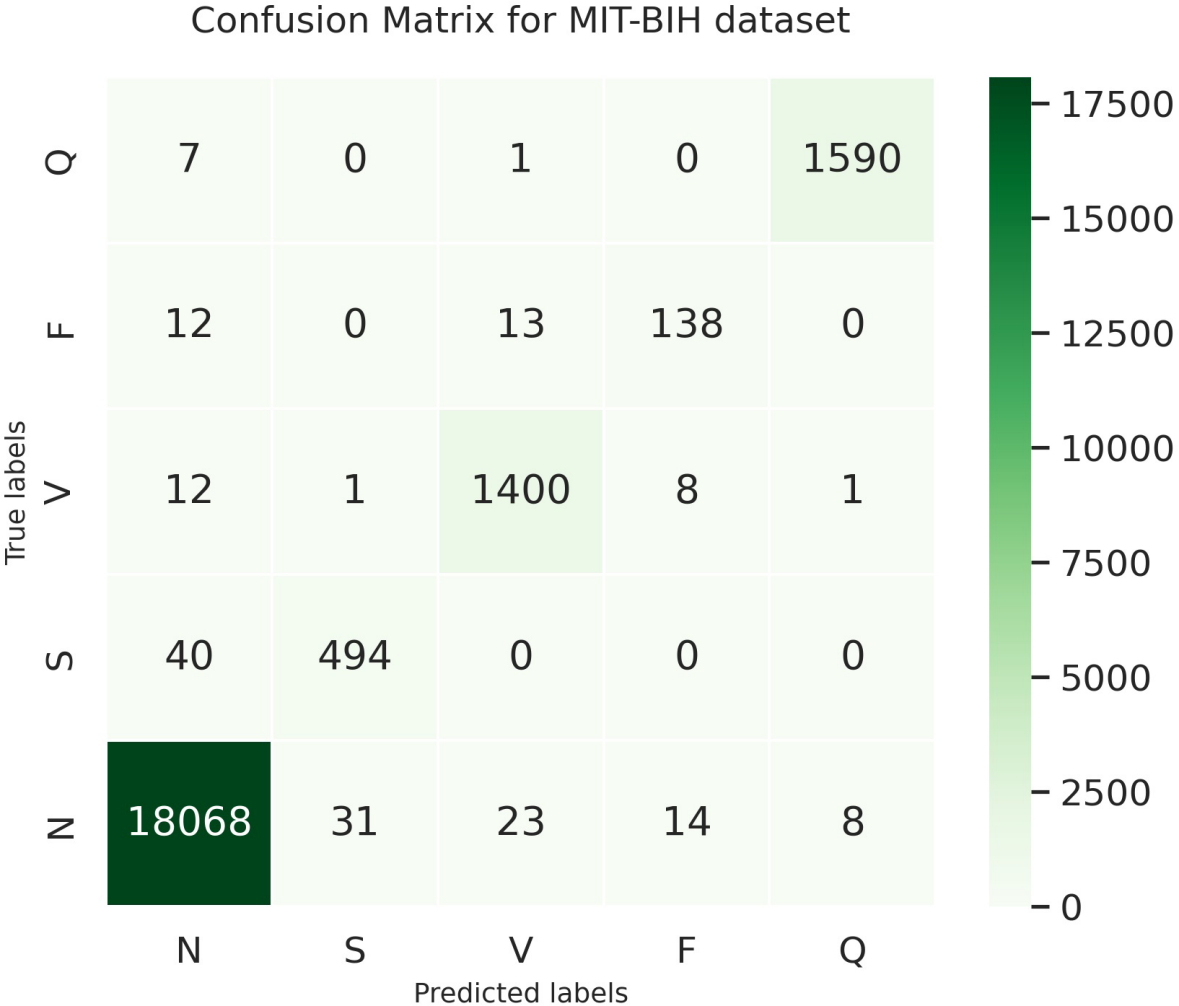
Confusion matrix for the proposed network on the Massachusetts Institute of Technology–Beth Israel Hospital (MIT-BIH) testing data.

**Figure 11:**
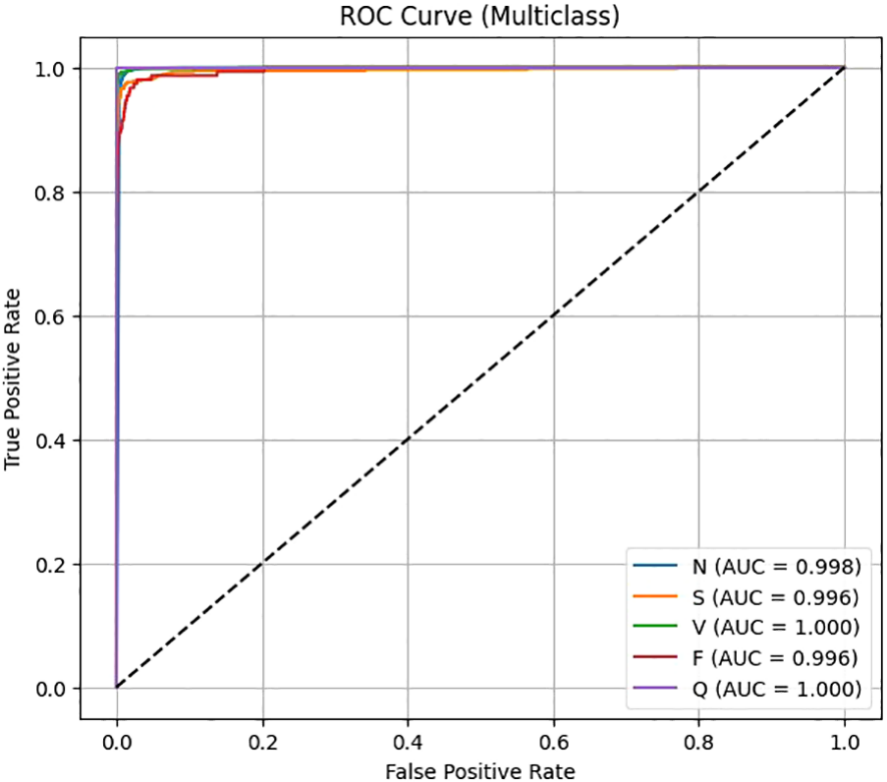
Receiver operating characteristic (ROC) curves of the proposed model evaluation on the Massachusetts Institute of Technology–Beth Israel Hospital (MIT-BIH) dataset.

The MIT-BIH dataset confusion matrix and class-wise classification results are listed in **Figure 10** and **Table 6**. The analysis demonstrated that 31 ‘S’-type beats were misclassified as ‘N’ and that 40 normal (‘N’) beats were misclassified as supraventricular (‘S’) beats. This suggests that normal and supraventricular beats are difficult for the model to differentiate, which is explained by the similarities in their morphological patterns. With only eight misclassifications out of 1,598 beats, the unclassifiable beats (‘Q’) had better accuracy and F1 scores due to their more recognizable morphological features. The data in **Table 6** indicate that the model consistently attained per-class accuracy of higher than 99.33%, illustrating its robust and reliable ability across all heartbeat categories. The proposed model outcomes on the normal class (‘N’) remained robust when class balancing was not applied. However, the sensitivity for the fusion class (‘F’) decreased, indicating that the limited number of F samples is a major issue. The model achieved sensitivity values of 99.58%, 92.51%, 98.45%, 88.67%, and 99.50% for the N, V, S, F, and Q classes, respectively. The reduced sensitivity of the F class highlights the difficulty of enhancing detection for minority classes in cardiac arrhythmia detection. Compared with the other categories, slight reductions in the sensitivity and the F1 scores were observed in the S and F classes. The discrepancy in performance is mainly due to the limited number of samples for these classes in the original dataset, which emphasizes the impact of data imbalance on classification accuracy. **Figure 11** shows the ROC curves for all five cardiac arrhythmia classes.

**Table 6:** Class-wise classification performance of the proposed model on the MIT-BIH testing data (imbalanced).

Class	AUC (%)	Sensitivity (%)	Specificity (%)	F1 score (%)	Accuracy (%)
N	99.75	99.58	98.10	99.60	99.33
S	99.63	92.51	99.85	93.21	99.67
V	99.99	98.45	99.82	97.94	99.73
F	99.68	88.67	99.90	85.45	99.79
Q	99.99	99.50	99.97	99.46	99.93
Overall	99.80	95.75	99.53	95.14	99.69

MIT-BIH, Massachusetts Institute of Technology–Beth Israel Hospital; AUC, area under the curve; N, normal; S, supraventricular ectopic; V, ventricular ectopic; F, fusion; Q, unknown.

The corresponding AUC values demonstrated excellent classification performance: 99.75% for the N class, 99.63% for S, 99.99% for V, 99.68% for F, and 99.99% for Q. The ROC curves for all classes were close to 1, indicating a high true positive rate with a low false-positive rate across all classes. An AUC value approaching 1 represents strong discriminative capability, confirming that the proposed model effectively distinguishes between the different arrhythmia categories. Notably, the V and Q classes achieved an AUC value of 99.99%, highlighting superior separability, while the remaining classes also exhibited highly competitive performance.

Overall, the high AUC values across all classes validate the robustness and reliability of the proposed model. These findings, together with the accuracy, loss, and confusion matrix analyses, provide a comprehensive evaluation of the proposed model performance and confirm its effectiveness for multiclass ECG classification.

### 3.3 Effect of class balancing

Majority of the existing ECG arrhythmia classification studies still encounter difficulties in achieving reliable prediction performance for the minority “F” class. In the MIT-BIH database, the “F” class contains only 0.733% of the total samples (802 out of 109,305 samples). This severe class imbalance significantly affects the learning capability of DL models, particularly for minority heartbeat categories. Therefore, class-balancing techniques are widely used to improve minority class representation during training. However, these techniques increase the number of minority class samples and the intrinsic variability of the data remains limited, thereby making accurate classification of the “F” class particularly challenging. For example, the framework proposed by Rafi and Ko (2022) achieved only 71.76% accuracy for the “F” class and 78.45% accuracy for the “S” class.

To evaluate the effect of class imbalance on the proposed network, experiments were conducted using both imbalanced and balanced training datasets. When trained on the original imbalanced dataset, the proposed model achieved an F1 score of only 79.65% for the minority “F” class, whereas the balanced training strategy improved the F1 score to 85.45%. Furthermore, the F1 scores of the other minority classes decreased by nearly 1%–5% under the imbalanced training condition. These observations demonstrate that class balancing plays a significant role in improving minority class prediction and overall model generalization.

In addition, different balancing techniques were evaluated to analyze their influence on the classification performance. **Table 7** summarizes the classification performance of different balancing techniques in terms of sensitivity, specificity, F1 score, and accuracy. Among the evaluated methods, SMOTE–Tomek achieved the highest classification accuracy and F1 score while maintaining superior minority class prediction capability. Although the class-weighted loss technique improved the sensitivity and specificity, its macro-F1 score remained comparatively lower, indicating reduced effectiveness in predicting minority heartbeat classes. In contrast, the improved performance of SMOTE–Tomek can be attributed to the combined effect of synthetic minority oversampling and Tomek link-based removal of overlapping samples, which enhances class separability and removes noisy instances during training. The proposed hybrid model trained using the SMOTE–Tomek technique achieved the highest F1 score and accuracy compared with the other class-balancing techniques.

**Table 7:** Comparison of different class-balancing techniques.

Technique	Sensitivity (%)	Specificity (%)	F1 score (%)	Accuracy (%)
SMOTE-ENN	94.97	99.44	94.75	99.53
ADASYN	94.81	99.39	94.63	99.42
Class-weighted loss	95.01	99.50	91.75	99.36
SMOTE–Tomek	95.75	99.53	95.14	99.69

SMOTE-ENN, synthetic minority oversampling technique edited nearest neighbor; ADASYN, adaptive synthetic.

### 3.4 Hyperparameter tuning

To fine-tune the proposed network and its key hyperparameters, a random search approach was employed, focusing on variables including convolutional filter sizes and numbers, GRU layers, and other crucial design aspects. At each step, key performance indicators, including accuracy, training time, total number of parameters, validation accuracy, and validation loss, were consistently monitored. The proposed framework attained the highest training and validation accuracy values of 99.99% and 99.63%, respectively, and the lowest training and validation losses of 0.0006% and 0.047%, respectively, on the INCART dataset. Comparative analysis of the proposed model with baseline models in terms of total parameters, training time, accuracy, and loss is presented in **Table 8**, which indicates that the proposed hybrid model has fewer parameters, achieving high accuracy and minimal loss during training and testing when compared with baseline models.

**Table 8:** Comparative analysis of the proposed model with baseline models in terms of total parameters, training time, accuracy, and loss.

Model	Total parameters	Train time (s/epoch)	Minimum loss (%)		Maximum accuracy (%)	
			Training	Validation	Training	Validation
CNN	597,381	17	0.0030	0.065	99.98	99.43
CNN+CA	597,381	16	0.0012	0.058	99.98	99.48
CNN+LSTM	622,725	28	0.0020	0.055	99.48	99.44
CNN+GRU	578,053	27	0.0009	0.050	99.99	99.46
CNN+Transformer	1,189,637	31	0.0043	0.056	99.89	99.57
Proposed	578,053	27	0.0006	0.047	99.99	99.63

CNN, convolutional neural network; CA, channel attention; LSTM, long short-term memory; GRU, gated recurrent unit.

### 3.5 Computational complexity analysis

The computational complexity of the key components in the proposed hybrid architecture, including 1D-Conv, CA, and GRU, is summarized in **Table 9**. This analysis employed the proposed model for each layer’s complexity, sequential process, and maximum path length. As presented in **Table 9**, majority of the layers in the proposed framework, including 1D-Conv, max pooling, BN, CA, and dense layers, support parallel computation with O(1) sequential processing, enabling effective hardware utilization. The recurrent architecture GRU requires O(n) sequential operations due to its recurrent nature, which introduces temporal dependencies across the input sequence. The 1D-Conv layers exhibit a computational complexity of $O(k\cdot n\cdot d^{2})$, enabling efficient extraction of local spatial features with shorter receptive paths. The BN and max pooling layers have linear complexity O(n.d) and constant path length, providing less computational overhead. The CA module has a marginal overhead of $O(n.d+d^{2}/r)$ to improve feature restoration through adaptive channel-wise weighting. The GRU has a computational complexity of $O(n.d^{2})$ and a maximum path length of O(n), enabling effective modeling of temporal dependencies within the ECG signal. Although this introduces sequential computation, it provides strong temporal learning capability that complements parallel feature extraction of convolutional layers.

**Table 9:** Computation complexity of the different layers in the proposed model.

Layer	Layer complexity	Sequential process	Maximum path length
1D-Conv	$O(k.n.d^{2})$	O(1)	$O({\text{log}}_{k}(n))$
Batch Norm.	O(n.d)	O(1)	O(1)
Max.Pooling	O(n.d)	O(1)	O(n)
Channel attention	$O(n.d+d^{2}/r)$	O(1)	O(1)
Gated recurrent unit	$O(n.d^{2})$	O(n)	O(n)
Dense layer	$O(d^{2})$	O(1)	O(1)

*n* denotes the input sequence length, *k* represents the convolution kernel size, *r* is the reduction ratio used in channel attention, and *d* indicates model dimension.

Overall, the proposed GRU-based hybrid network extracts both local morphological patterns and temporal dependencies in the ECG signal. While the integration of GRU increases the computational cost compared with purely convolutional architectures, the model maintains a favorable balance between computational efficiency and achieves representational capability with a reduced path length and parallel computation.

### 3.6 Validation with the INCART database

**Figure 12:**
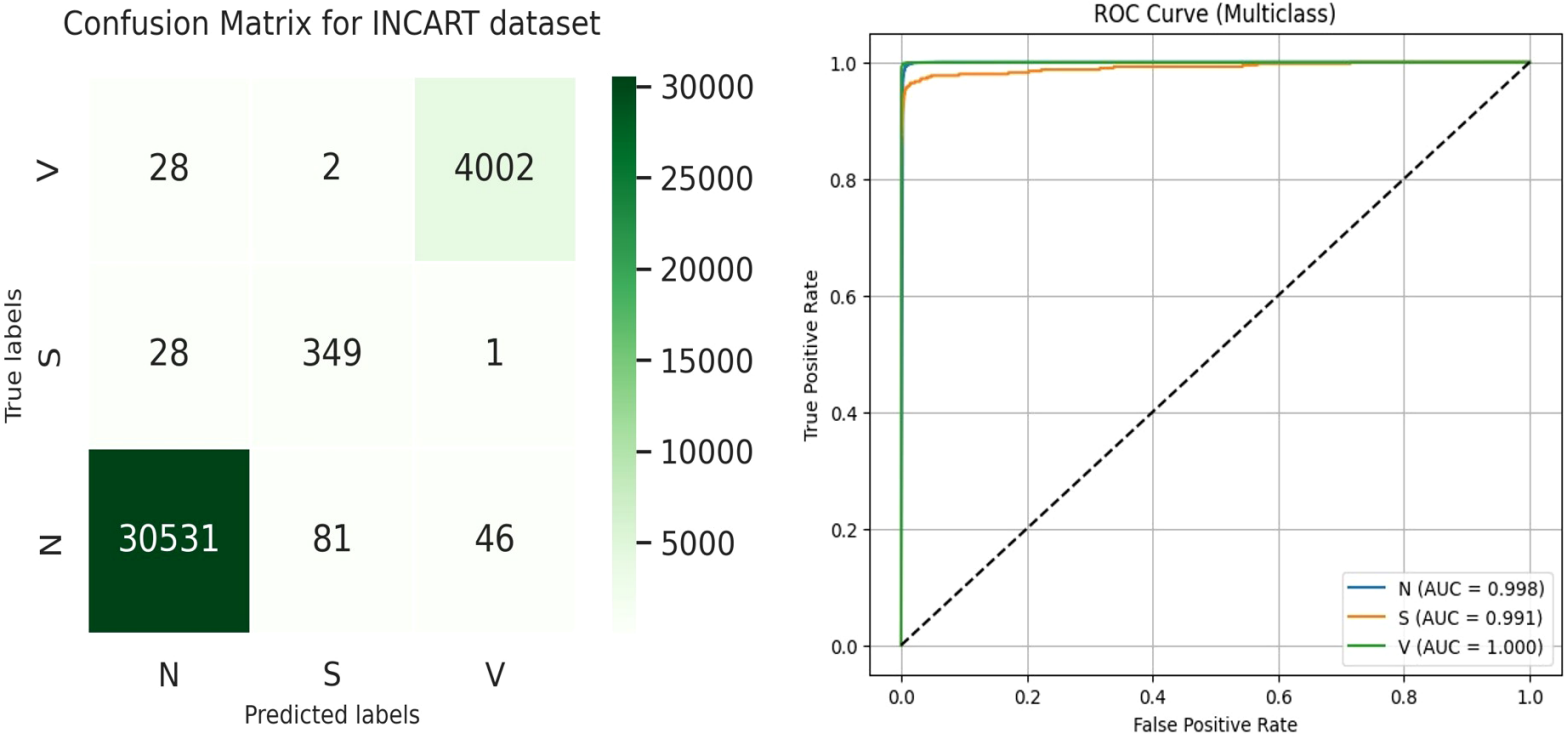
Confusion matrix and receiver operating characteristic (ROC) curves of the proposed model validated on the INCART dataset.

The proposed model was further validated using the INCART database to comprehensively evaluate its generalization capability, robustness, and reliability. The model attained an outstanding accuracy of 99.73% and an F1 score of 96.79% for the three-class arrhythmia classification task. The confusion matrix and the ROC curves for three cardiac arrhythmia classes are shown in **Figure 12**, while the per-class performance metrics results are summarized in **Table 10**. The performance on the INCART dataset was superior to that on the MIT-BIH dataset in terms of F1 score and accuracy, indicating the reliable and robust performance of the proposed network. The proposed hybrid model effectively reduces dataset bias by capturing both local and global temporal features from the ECG signals, ensuring strong generalization. The proposed model exhibited robustness and dataset-invariant characteristics, demonstrating its potential for implementation in real-world clinical settings.

**Table 10:** Class-wise classification performance of the proposed model on the INCART imbalanced testing data.

Class	AUC (%)	Sensitivity (%)	Specificity (%)	F1 score (%)	Accuracy (%)
N	99.89	99.69	98.90	99.76	99.59
V	99.18	92.59	99.90	91.62	99.82
S	99.99	99.36	99.83	99.01	99.77
Overall	99.69	97.21	99.54	96.79	99.73

AUC, area under the curve; N, normal; V, ventricular ectopic; S, supraventricular ectopic.

### 3.7 Generalization on the MIT-BIH SVDB dataset

**Figure 13:**
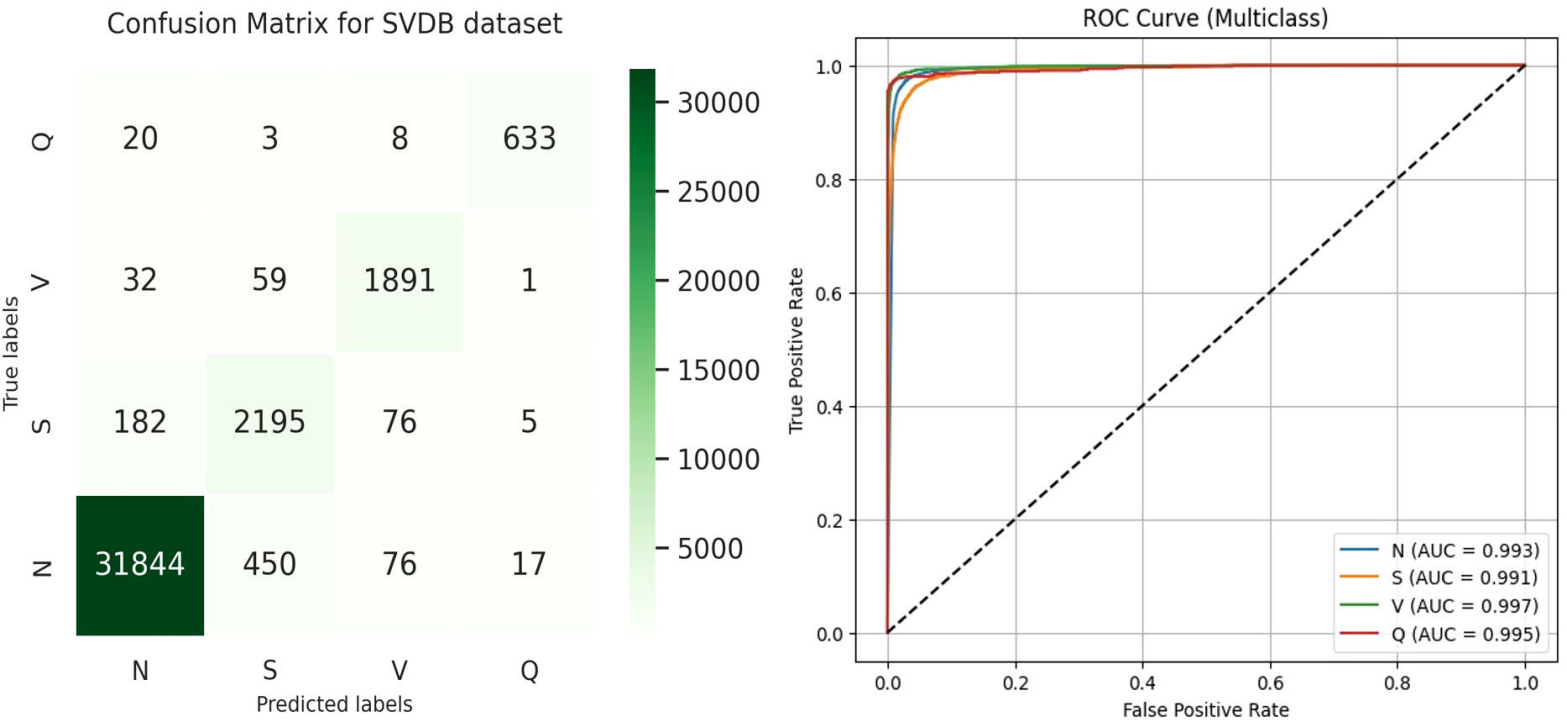
Confusion matrix and receiver operating characteristic (ROC) curves of the proposed network on the MIT-BIH Supraventricular Arrhythmia Database (SVDB) testing data.

The confusion matrix and the ROC curves for four cardiac arrhythmia classes are shown in **Figure 13**. The class-wise evaluation results for the MIT-BIH SVDB dataset are presented in **Table 11**. The results highlighted that the proposed framework achieved robust performance across all heartbeat categories, with class-wise accuracy values exceeding 97.93% and sensitivity values ranging from 95.33% to 98.32%. In particular, our hybrid model attained a sensitivity of 95.37% and a specificity of 99.55% on the ventricular arrhythmia class, suggesting that these heartbeats are morphologically distinct and much faster to classify. The high accuracy, sensitivity, specificity, F1 score, and AUC values exceeded 95%. These results confirm the high level of robustness and reliability of the proposed model within this study. In summary, the proposed model showed outstanding performance on the selected MIT-BIH SVDB data for validation, making the model reliable for the classification of various cardiac arrhythmia classes.

**Table 11:** Class-wise performance analysis of the proposed model on the SVDB imbalanced testing data.

Class	AUC (%)	Sensitivity (%)	Specificity (%)	F1 score (%)	Accuracy (%)
N	99.25	98.32	95.92	98.80	97.93
S	99.10	89.31	98.84	84.99	97.93
V	99.71	95.37	99.55	93.75	99.33
Q	99.49	95.33	99.94	95.91	99.86
Overall	99.39	94.58	98.66	93.36	98.77

SVDB, MIT-BIH Supraventricular Arrhythmia Database; AUC, area under the curve; N, normal; S, supraventricular ectopic; V, ventricular ectopic; Q, unknown.

### 3.8 Ablation study

**Figure 14:**
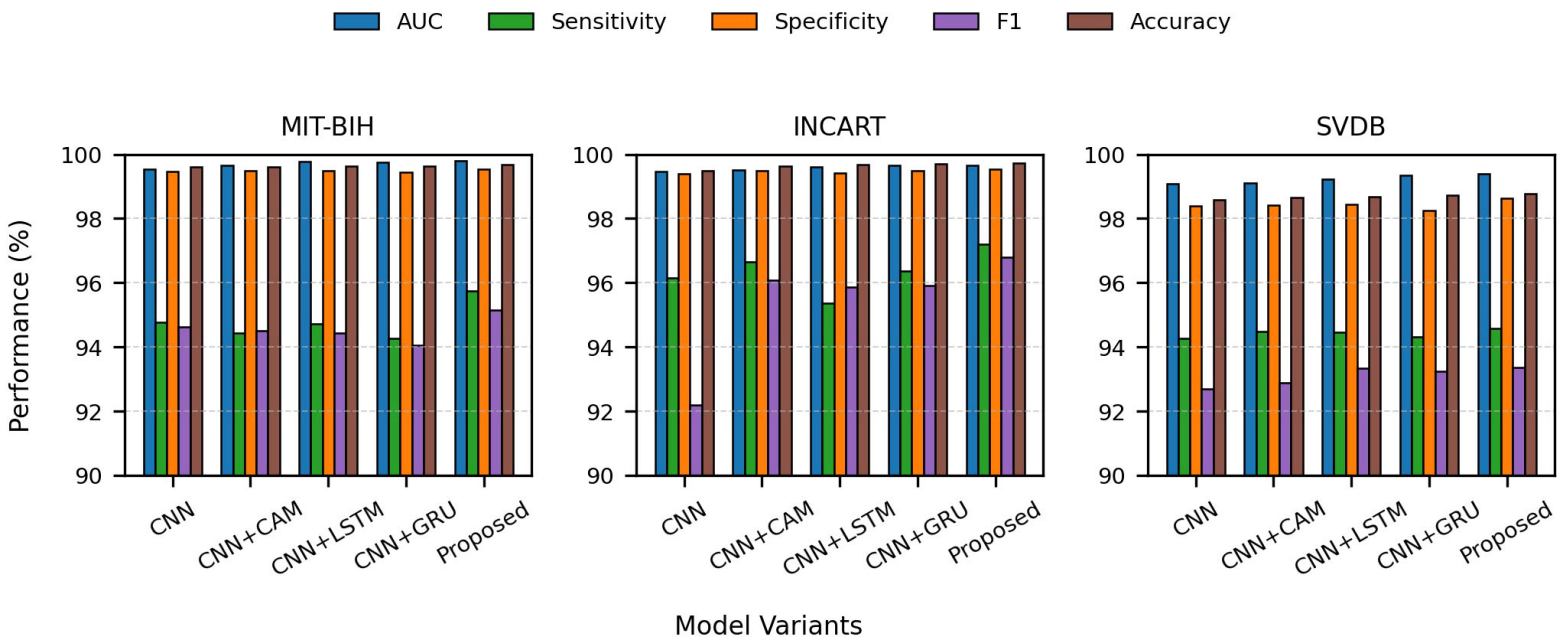
Performance comparison of the convolutional neural network (CNN), channel attention (CA), long short-term memory (LSTM), and gated recurrent unit (GRU) model combinations on the Massachusetts Institute of Technology–Beth Israel Hospital (MIT-BIH), INCART, and MIT-BIH Supraventricular Arrhythmia Database (SVDB) datasets.

The ablation study results on the MIT-BIH, INCART, and SVDB ECG datasets are presented in **Table 12**. The performance comparison of the CNN, CA, and GRU model combinations on the MIT-BIH, INCART, and SVDB datasets are shown in **Figure 14**, evaluating the combinations of three key components of the proposed hybrid framework: CNN, CA, and GRU. The baseline hybrid models such as the CNN, LSTM, and GRU models achieved accuracy values of 99.60%, 99.62%, 99.65% and F1 scores of 94.63%, 94.43%, and 94.06%, respectively, on the MIT-BIH dataset, indicating efficient spatial–temporal feature extraction. Integrating CA with CNN increased the sensitivity from 96.14% to 96.66% and improved the F1 score from 92.20% to 96.07% on the INCART database, demonstrating the capability of CA to emphasize informative feature representation. The performance was further enhanced by incorporating GRU with CNN, achieving an accuracy of 98.72% and a specificity of 98.25% on the SVDB database, representing effectiveness in capturing temporal features. The proposed model integrating GRU with CNN and CA achieved an overall optimal performance when analyzed with the MIT-BIH database, with 95.75% sensitivity, 99.53% specificity, 95.14% F1 score, and 99.69% accuracy. These findings confirm that each component makes a substantial contribution to performance and that, when combined, they produce a more accurate and reliable classification of ECG arrhythmias.

**Table 12:** Ablation study results of the convolutional neural network (CNN), channel attention (CA), long short-term memory (LSTM), and gated recurrent unit (GRU) model combinations on the Massachusetts Institute of Technology–Beth Israel Hospital (MIT-BIH), INCART, and MIT-BIH Supraventricular Arrhythmia Database (SVDB) datasets.

Dataset	Model variant	AUC (%)	Sensitivity (%)	Specificity (%)	F1 score (%)	Accuracy (%)
MIT-BIH	CNN	99.53	94.76	99.47	94.63	99.6
	CNN+CA	99.65	94.44	99.48	94.51	99.62
	CNN+LSTM	99.78	94.72	99.49	94.43	99.62
	CNN+GRU	99.75	94.26	99.45	94.06	99.65
	Proposed	99.8	95.75	99.53	95.14	99.69
INCART	CNN	99.46	96.14	99.39	92.2	99.49
	CNN+CA	99.51	96.66	99.48	96.07	99.64
	CNN+LSTM	99.6	95.37	99.42	95.86	99.69
	CNN+GRU	99.65	96.37	99.49	95.9	99.7
	Proposed	99.65	97.21	99.54	96.79	99.73
SVDB	CNN	99.08	94.26	98.39	92.69	98.58
	CNN+CA	99.1	94.48	98.41	92.89	98.66
	CNN+LSTM	99.23	94.46	98.43	93.34	98.69
	CNN+GRU	99.34	94.32	98.25	93.23	98.72
	Proposed	99.39	94.58	98.63	93.36	98.77

### 3.9 Statistical significance

To validate the effectiveness of the proposed GRU hybrid model, we conducted a statistical significance test analysis. The baseline CNN and LSTM models and the proposed model were evaluated with multiple runs, with the performance metrics under evaluation including specificity, sensitivity, accuracy, and the F1 score. A two-tailed paired *t*-test was conducted to analyze the proposed GRU hybrid model with its baseline model variants. The *p*-values measured from the *t*-tests for all evaluation metrics are summarized in **Table 13**. The *p*-values measured from all baseline and proposed model combination comparisons were consistently <0.05, indicating that the performance gain attained by our CNN+CA+GRU model was highly statistically significant across the CNN and LSTM baseline model comparisons, further indicating that the integration of GRU with CA significantly improved the predictive ability of the hybrid GRU model. The low *p*-values indicate that the observed improvements are statistically significant and not due to random variation. These results confirmed the consistently robust and reliable performance of the proposed hybrid GRU model over several runs. This evaluation confirms the model’s capability to capture both local morphological and global temporal dependencies, emphasizing its reliability for consistent and accurate ECG cardiac arrhythmia classification in clinical healthcare settings.

**Table 13:** Paired *t*-test results for statistical significance.

Comparison	Sensitivity *p*-value	Specificity *p*-value	F1 score *p*-value	Accuracy *p*-value
CNN+CA+GRU *vs*. CNN	0.039	0.002	0.041	0.01
CNN+CA+GRU *vs*. CNN+CA	0.035	0.005	0.032	0.01
CNN+CA+GRU *vs*. CNN+LSTM	0.043	0.037	0.045	0.02
CNN+CA+GRU *vs*. CNN+GRU	0.010	0.002	0.030	0.04

CNN, convolutional neural network; CA, channel attention; GRU, gated recurrent unit; LSTM, long short-term memory.

### 3.10 Comparison with existing techniques

To establish the robustness and clinical suitability of the proposed framework, a comprehensive comparative analysis was conducted against recent DL models using the widely adopted INCART and MIT-BIH datasets. The comparison included recent architectures such as CNN, CNN-RNN, CNN-LSTM, CNN with attention mechanisms, residual network (ResNet)-based models, and CNN–transformer frameworks. For evaluation, only those models using similar class definitions, datasets, and evaluation metrics were included for comparison. Performance was assessed using key metrics including sensitivity, precision, specificity, accuracy, and the F1 score, as summarized in **Tables 14**, **15**.

**Table 14:** Comparison of the proposed model with existing techniques on the INCART database.

Reference	Class	Model	Accuracy (%)	F1 (%)	Sensitivity (%)	Precision (%)	Specificity (%)
Peimankar et al. (2024)	5	ResNet	98	98	98	98	–
Pandey et al. (2025b)	5	M2AM-Deep BiLSTM	93.82	–	94.34	96.20	–
Gao et al. (2025)	3	SE-ConvNet	95.18	96.39	96.41	96.37	92.70
Issa et al. (2025)	3	Hybrid DNN-EMD	99.38	–	99.69	99.33	99.38
Yu et al. (2025)	4	1D CNN+Attn (OOD)	98.15	97.34	98.15	97.57	–
Pandey et al. (2024)	4	M2MASC CNN-BiLSTM	97.85	–	98.16	97.53	–
Pandey et al. (2025a)	4	SCN-Deep BiLSTM	97	97	98	97	–
Li et al. (2022)	3	SE-ResNet	98.71	–	99.12	–	99.59
Mokhtari et al. (2025)	3	PMAT+2D-CNN	98.37	79.37	–	–	–
Hassan et al. (2022)	5	CNN-BiLSTM	95	80.4	92.2	73.8	92.3
Romdhane and Pr (2020)	5	Deep CNN	97.07	96.98	98.41	–	98.37
Islam et al. (2024)	3	CNN+Transformer (CAT-Net)	99.58	96.15	96.86	–	99.48
Elsheikhy et al. (2025)	5	CNN+CAM	99.48	81.84	77.38	99.4	99.37
De Waele et al. (2026)	3	CNN+Transformer	94.5	76.8	93.3	71.1	–
Proposed (INCART)	3	CNN+CA+GRU	99.73	96.79	97.21	99.5	99.54

CNN, convolutional neural network; CA, channel attention; GRU, gated recurrent unit; LSTM, long short-term memory; M2AM, modified multiclass attention mechanism; SE-ConvNet, squeeze-and-excitation convolutional neural network; DNN, deep neural network; EMD, empirical mode decomposition; BiLSTM, bidirectional LSTM; PMAT, progressive moving average transform; SE-ResNet, squeeze-and-excitation residual network; M2MASC, modified mixed attention-enabled search optimizer; CAT-Net, convolution, attention, and transformer network; CAM, class activation mapping.

**Table 15:** Comparison of the proposed model with existing techniques on the Massachusetts Institute of Technology–Beth Israel Hospital (MIT-BIH) dataset.

Reference	Class	Model	Accuracy (%)	F1 (%)	Sensitivity (%)	Precision (%)	Specificity (%)
Qi et al. (2024)	5	Hybrid ResNet	99.09	96.33	97.56	95.18	–
Arora et al. (2023)	5	2D CNN+LSTM	96.2	91.8	96.9	87.2	98.6
Wang (2021)	5	CNN+GRU	97.9	–	98	–	97.8
Lamba et al. (2025)	5	BiLSTM+FCN	99.1	98.8	98.9	99.1	–
Shah et al. (2024)	5	Transformer+CovNet	98.6	98.4	98.6	97.9	97.8
Khan et al. (2023)	5	1D-CNN+ResNet+SMOTE	98.63	92.63	92.41	92.86	99.06
Daydulo et al. (2023)	5	ResNet-50	99.2	99.2	99.2	–	99.5
Shoughi et al. (2024)	5	CNN+BiLSTM+SMOTE	98.53	92.94	92.94	92.24	99.5
Anand et al. (2024)	5	ResNet-18	98.14	97.3	97.3	96.5	–
Sun et al. (2024)	5	CNN+LSTM+SE	98.5	98	98	97	–
Bahrami and Fotouhi (2025)	4	DCNN	99.13	93.64	92.28	95.04	–
Tenepalli and Navamani (2025a)	5	EGOLNNet	99.6	99.5	99.4	99.7	99.5
Keskes et al. (2022)	2	RF classifier	89.36	94.30	89.36	90.06	95
Jin et al. (2023)	2	LSTM	94	93.52	97.59	–	97.97
Guo et al. (2019)	5	DenseNet+GRU	92	–	82	–	96
Essa and Xie (2021)	4	CNN+LSTM	95.81	71.06	69.20	–	94.56
Mohammed et al. (2026)	5	CNN+LSTM+GRU	97	97	–	92	–
Zhao et al. (2024)	4	TCN+ResNet	97	87	92	92	–
He et al. (2025)	5	CANet	94.41	79.02	92.41	87.23	–
Panwar et al. (2025)	5	1D-CNN	99.3	97.96	97.96	99	99.1
Wu et al. (2026)	5	GNN+Transformer	98.27	97.76	98.27	98.08	–
Islam et al. (2024)	3	CNN+Transformer (CAT-Net)	99.14	94.69	94.65	–	99.35
Ikram et al. (2025)	5	Transformer+PCA	97.1	95	–	–	–
Alghieth (2025)	5	CNN+Transformer	98.2	96.4	95.3	98.2	–
Niloy et al. (2025)	5	MuRe-Lead Aware Transformer	94	88.4	85.68	85.9	–
Liu (2025)	5	CNN-BERT	95.08	94.91	94.69	94.92	99.32
El-Ghaish and Eldele (2024)	5	ECGTransForm	99.35	94.26	93.15	95.47	–
Proposed (MIT-BIH)	5	CNN+Channel Attn.+GRU	99.69	95.14	95.75	99.22	99.53

ResNet, residual network; CNN, convolutional neural network; LSTM, long short-term memory; GRU, gated recurrent units; BiLSTM, bidirectional long short-term memory; FCN, fully convolutional network; SMOTE, synthetic minority oversampling technique; DCNN, deep convolutional neural network; RF, random forest; TCN, temporal convolutional network; CAT-Net, convolution, attention, and transformer network; GNN, graph neural network; PCA, principal component analysis.

The proposed model was first validated on the St. Petersburg INCART database for three-class classification. As shown in **Table 14**, the model achieved an accuracy of 99.73% and an F1 score of 96.79%, outperforming the majority of the existing approaches. Compared with earlier works, Peimankar et al. (2024) reported 98.00% accuracy using ResNet, while Yu et al. (2025) achieved 98.15% accuracy with a 1D CNN–attention model. Pandey et al. (2025b, 2024) proposed BiLSTM-based hybrid models with accuracy values of 93.82% and 97.85%, but rely on multi-lead ECG signals and complex fusion strategies. Li et al. (2022) and Issa et al. (2025) reported competitive performance using SE-ResNet and hybrid DNN models. However, these techniques either lack complete evaluation metrics or depend on multi-lead inputs. Similarly, Islam et al. (2024) achieved high accuracy using CAT-Net, but with increased architectural complexity. Hassan et al. (2022) proposed a CAT-BiLSTM model integrated with convolutional and bidirectional LSTM layers, achieving 95% accuracy. However, the model exhibited relatively lower F1 score and precision, indicating limitations in handling class imbalance. Romdhane and Pr (2020) developed a deep CNN-based approach with focal loss to address class imbalance, achieving 97.07% accuracy and 96.98% F1 score; nevertheless, the model relies solely on spatial feature extraction without explicitly modeling temporal dependencies. Elsheikhy et al. (2025) proposed a lightweight CNN-integrated CA framework and achieved 99.48% accuracy. However, the model reported a lower sensitivity of 77.38% and an F1 score of 81.84%, representing limited effectiveness in minority heartbeat class classification. Similarly, De Waele et al. (2026) proposed a CNN–transformer-based framework for heartbeat classification, achieving strong sensitivity performance. However, the lower F1 score and precision indicate limitations in maintaining balanced classification performance across minority heartbeat classes. In contrast, the proposed model achieved high performance while utilizing a single-lead ECG signal, demonstrating both efficiency and effectiveness.

To further evaluate generalization capability, the proposed model was tested on the widely used MIT-BIH database for cardiac arrhythmia classification. As presented in **Table 15**, the proposed model achieved an accuracy of 99.69% and an F1 score of 95.14% for five-class classification, outperforming the most recent state-of-the-art methods. Qi et al. (2024) developed a hybrid ResNet model and achieved 99.09% accuracy, while Lamba et al. (2025) reported 99.10% accuracy using a BiLSTM-FCN framework with higher computational costs. Transformer-based approaches such as TransCovNet (Shah et al., 2024) and GNN+Transformer (Wu et al., 2026) demonstrate competitive performance, but require significantly higher computational resources and suffer from high complexity. Tenepalli and Navamani (2025a) reported superior performance with 99.6% accuracy and 99.5% F1 score using EGOLNNet; however, such architectures are typically deeper, more complex, and have higher computational requirements. Similarly, Daydulo et al. (2023) achieved 99.2% accuracy using a ResNet-50 model, which also increases computational overhead. Panwar et al. (2025) obtained 99.3% accuracy using a 1D-CNN model, but with their model exhibiting a slightly lower performance on other performance metrics compared with the proposed framework.

Several hybrid models, including CNN-LSTM architectures (Arora et al., 2023; Essa and Xie, 2021; Mohammed et al., 2026), CNN+BiLSTM, and CNN+ResNet with SMOTE (Shoughi et al., 2024; Khan et al., 2023), demonstrate moderate performance, but often suffer from reduced F1 scores due to imbalanced class-wise results. Similarly, Sun et al. (2024) and Anand et al. (2024) introduced a hybrid DL model, which attained accuracy values of 98.5% and 98.14%, respectively, but demonstrate comparatively lower robustness across multiple evaluation metrics. Other techniques such as TCN-ResNet (Zhao et al., 2024), CANet (He et al., 2025), and DenseNet+GRU (Guo et al., 2019) achieve moderate performance, but suffer from computational overhead and exhibit lower sensitivity and lack of balanced evaluation across all metrics. In addition, classical and lightweight models, including the RF classifier (Keskes et al., 2022) and LSTM-based approach (Jin et al., 2023), have shown significantly lower performance, indicating their limitations in handling complex ECG patterns. Alghieth (2025) proposed a CNN–transformer-based architecture for ECG anomaly detection, achieving competitive classification performance, whereas El-Ghaish and Eldele (2024) introduced ECGTransForm using a bidirectional transformer architecture with 99.35% accuracy. Although transformer-based networks exhibit strong feature learning capability, they often require increased computational resources and complex optimization strategies. Furthermore, Niloy et al. (2025) proposed a lead-aware multi-resolution transformer framework for beat-level ECG classification, achieving comparatively lower F1 score and sensitivity, indicating limitations in minority class prediction. Similarly, Liu (2025) developed a hybrid CNN-BERT architecture for ECG arrhythmia classification, achieving strong performance, but with comparatively lower overall classification accuracy than the proposed framework. Furthermore, Ikram et al. (2025) developed a hybrid transformer model, achieving an accuracy of 97.1% and an F1 score of 95%, but did not report complete performance metrics, which restricts clinical interpretability. In contrast, the proposed model consistently provides balanced performance across all key performance metrics.

The competitive performance of the proposed hybrid framework can be attributed to the effective integration of spatial feature extraction using CNN, temporal dependency modeling through GRU, and enhanced feature representation using CA. Furthermore, the integration of the SMOTE–Tomek technique effectively addresses class imbalance, improving the classification performance across minority classes. Overall, the proposed model achieved 99.73% accuracy on the INCART dataset (three-class), 98.77% on the SVDB dataset (four-class), and 99.69% on the MIT-BIH dataset (five-class), demonstrating strong generalization capability across datasets with varying characteristics. Compared with existing methods, the proposed approach offers a favorable trade-off between accuracy, computational efficiency, and model complexity. In addition, the integration of the Grad-CAM module enhances interpretability by providing visual explanations of model predictions, highlighting clinically relevant ECG regions. This further strengthens the reliability and practical applicability of the proposed framework in real-world clinical and wearable healthcare environments.

### 3.11 Explanation of the model predictions using Grad-CAM

The proposed GRU-based hybrid DL network for ECG cardiac arrhythmia classification integrates a Grad-CAM module to improve the interpretability of the model predictions. This strategy enables visualization of the most influential temporal regions within the ECG signal that contribute to the decision-making process. The model exhibited high classification performance on the INCART, MIT-BIH, and SVDB datasets, achieving higher accuracy, specificity, sensitivity, and F1 score in the detection of various ECG arrhythmias. Beyond quantitative performance, the integration of Grad-CAM improves the transparency of the model by emphasizing diagnostically relevant ECG waveform segments. To ensure temporal consistency, each ECG recording signal was segmented into fixed-length heartbeat windows of 300 samples (91 samples prior and 201 samples after) to the R-peak locations. This alignment technique preserves complex morphological features such as the P-wave, QRS complex, and T-wave across all heartbeat samples. In addition, amplitude normalization was applied to minimize inter-patient variability and highlight the morphological characteristics over the absolute ECG signal magnitude.

**Figure 15:**
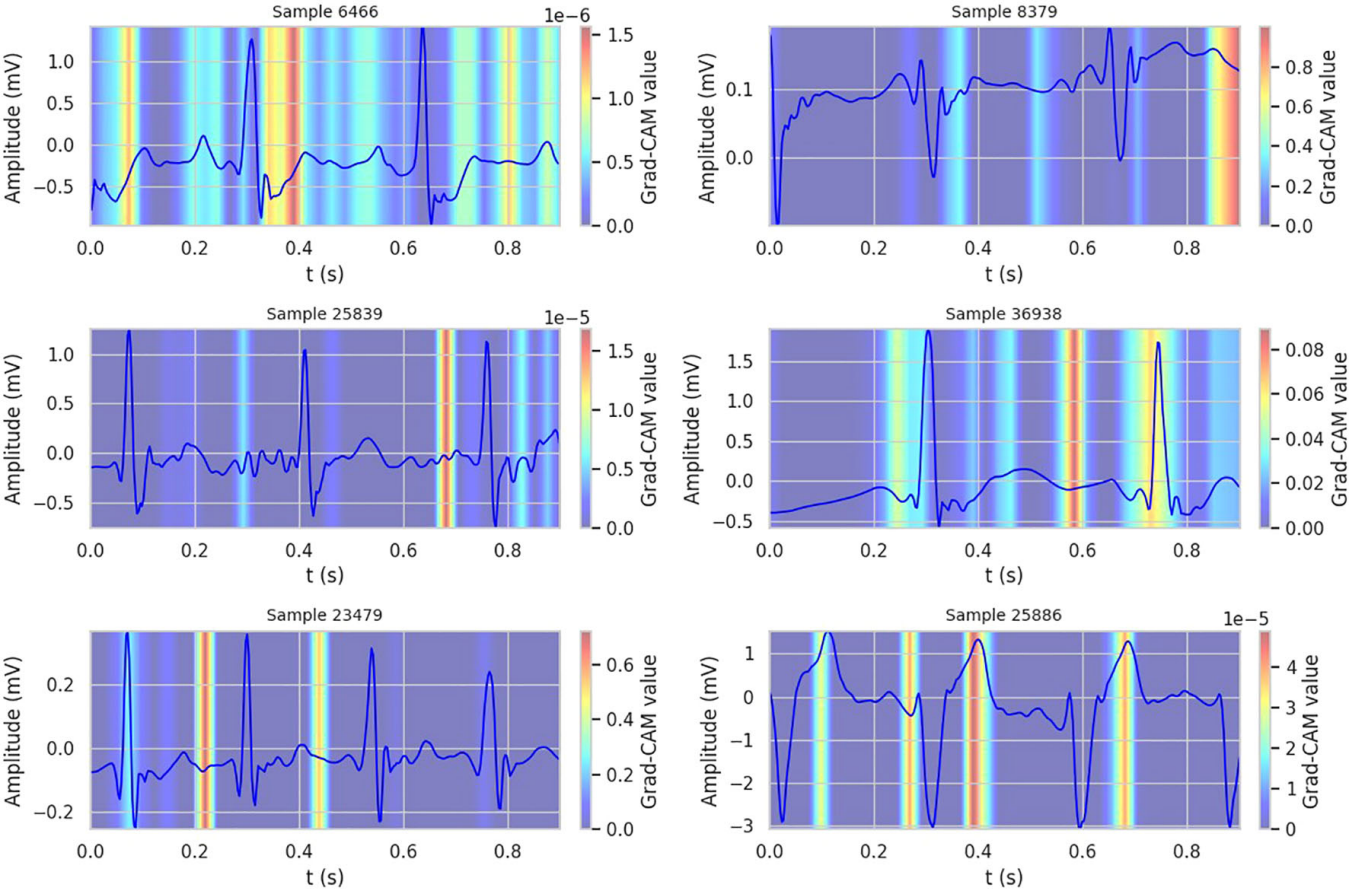
Multiple raw electrocardiogram (ECG) samples with overlaid gradient-weighted class activation mapping (Grad-CAM) heatmaps, highlighting the most important portions for the model classification of cardiac conditions.

**Figure 15** demonstrates representative Grad-CAM visualizations produced by the proposed GRU hybrid model on test ECG samples. Each subplot illustrates an ECG signal waveform overlaid with a heatmap representing the relative importance of various temporal regions toward the classification result. Warmer colors correspond to higher Grad-CAM activation values, indicating a stronger influence on the classification results. In **Figure 15**, it is evident that the proposed model consistently focuses on clinically important regions of the ECG signal. In particular, stronger Grad-CAM activations are concentrated around QRS complex regions in abnormal heartbeat samples, indicating that the model effectively captures ventricular depolarization characteristics associated with arrhythmia detection. Additional activations are also observed around the P-wave and T-wave regions for specific heartbeat categories, demonstrating that the proposed framework utilizes comprehensive temporal ECG morphology for classification. To further validate the explainability analysis quantitatively, the normalized Grad-CAM activation distributions were analyzed across physiologically relevant ECG waveform regions, including the P-wave, QRS complex, and T-wave intervals. The ECG waveform regions were approximately segmented based on the R-peak aligned heartbeat windows adopted during preprocessing. The average Grad-CAM activation values for the different ECG regions are summarized in **Table 16**. The results demonstrated that the proposed model consistently assigns higher attention to the QRS complex region, which is clinically important for arrhythmia diagnosis. In addition, the class-wise Grad-CAM attention concentration around the QRS complex was analyzed to evaluate the consistency of clinically relevant feature localization across heartbeat categories. The average percentage of Grad-CAM activation concentrated around the QRS region for each heartbeat class is presented in **Table 17**. The quantitative analysis demonstrated that the proposed model consistently concentrates substantial Grad-CAM attention around clinically significant QRS regions across multiple heartbeat classes, indicating reliable localization of diagnostically relevant ECG features. The quantitative Grad-CAM evaluation confirms that the proposed framework learns physiologically meaningful ECG representations by consistently focusing on diagnostically relevant waveform regions during heartbeat classification. The consistency between Grad-CAM highlighted regions and known electrophysiological characteristics reinforces the reliability and clinical relevance of the proposed hybrid model.

**Table 16:** Average normalized gradient-weighted class activation mapping (Grad-CAM) activation across the electrocardiogram (ECG) waveform regions.

ECG region	Average Grad-CAM activation
P-wave	0.31
QRS complex	0.82
T-wave	0.47

**Table 17:** Average gradient-weighted class activation mapping (Grad-CAM) attention concentration around QRS regions.

Heartbeat class	Mean attention QRS (%)
N	78.4
S	74.1
V	81.7
F	69.5
Q	72.8

N, normal; S, supraventricular ectopic; V, ventricular ectopic; F, fusion; Q, unknown.

Furthermore, the Grad-CAM-based interpretability mechanism facilitates clinical decision support by mapping the learned deep features to interpretable ECG waveform components. This explainability capability improves the trustworthiness of the proposed automated arrhythmia classification framework and enhances its suitability for real-world clinical and wearable healthcare applications. Overall, the proposed CNN+Channel Attention+GRU framework combines high predictive performance with robust explainability, demonstrating its effectiveness for accurate and reliable ECG arrhythmia diagnosis.

## 4 Future directions

Although the proposed model demonstrates strong performance on benchmark datasets such as MIT-BIH, INCART, and SVDB using single-lead ECG signals, several enhancements can be explored to further improve its clinical applicability and robustness. Future work should focus on the collection and integration of large-scale, diverse, and multicenter ECG datasets, including the PTB-XL, Chapman–Shaoxing, and Georgia 12-lead ECG databases, to improve generalization across varying demographics, acquisition conditions, and noise environments. In addition, extending the framework to multi-lead ECG analysis could provide richer spatial information and enhance the detection of complex, transient, and lead-specific ECG arrhythmias. The integration of multi-label classification strategies would further enable the simultaneous detection of existing cardiac abnormalities, thereby enhancing diagnostic capability. Finally, comprehensive benchmarking against state-of-the-art machine DL models, along with optimization of the model architecture for computational efficiency, is essential for real-time deployment. These advancements collectively aim to improve the accuracy, scalability, and translational potential of the proposed system in real-world healthcare settings.

## 5 Conclusion

This work developed an explainable hybrid DL framework for accurate classification of cardiac arrhythmias using single-lead ECG signals. The proposed model integrates 1D-CNN, CA, and GRU to effectively extract both local morphological features and temporal dependencies. In addition, the integration of the Grad-CAM module improves model interpretability by emphasizing the most important portions of the ECG signal assisting classification decisions, thereby enhancing clinical trust and transparency.

The experimental outcomes demonstrated that the proposed hybrid network achieves superior performance across three benchmark datasets, namely, MIT-BIH, INCART, and SVDB, with classification accuracy values of 99.69%, 99.73%, and 98.77%, respectively. The model also attained high sensitivity, precision, specificity, and F1 scores, confirming its robustness and reliability. The proposed framework demonstrates strong potential for real-time deployment in wearable devices, remote monitoring systems, and Internet of things (IoT)-enabled healthcare applications due to its computational efficiency and use of single-lead ECG signals. Moreover, the explainability provided by Grad-CAM facilitates clinically meaningful insights by aligning model attention with relevant ECG waveform components, thereby supporting informed decision making.

Despite its promising performance, certain limitations remain. The evaluation was primarily conducted on publicly available datasets, which may not fully represent real-world clinical variability. In addition, the training process requires moderate computational resources, which may pose challenges for deployment in highly resource-constrained environments. Future work will focus on validating the proposed model on larger and more diverse clinical datasets, optimizing the architecture for low-power devices, and conducting prospective clinical studies to further enhance its applicability in real-world healthcare settings.

## Data Availability

The original contributions presented in the study are included in the article/supplementary material. Further inquiries can be directed to the corresponding author.
